# Assessing the quality of mobile applications in chronic disease management: a scoping review

**DOI:** 10.1038/s41746-021-00410-x

**Published:** 2021-03-10

**Authors:** Payal Agarwal, Dara Gordon, Janessa Griffith, Natasha Kithulegoda, Holly O. Witteman, R. Sacha Bhatia, Andre W. Kushniruk, Elizabeth M. Borycki, Lise Lamothe, Elena Springall, James Shaw

**Affiliations:** 1 Institute for Health Systems Solutions and Virtual Care, Women’s College Hospital, Toronto, ON Canada; 2 Department of Family and Community Medicine, University of Toronto, Toronto, ON Canada; 3 Institute of Medical Science, University of Toronto, Toronto, ON Canada; 4 Faculty of Health Sciences, Douglas College, New Westminster, BC Canada; 5 Institute for Health Policy, Management and Evaluation, University of Toronto, Toronto, ON Canada; 6 Department of Family and Emergency Medicine, Faculty of Medicine, Laval University, Québec City, QC Canada; 7 Office of Education and Continuing Development, Faculty of Medicine, Laval University, Québec City, QC Canada; 8 Center Recherche Sur Les Soins Et Les Services De Première Ligne De l’Université Laval CERSSPL-UL, Québec City, QC Canada; 9 Population Health and Optimal Health Practices, Research Centre of the CHU de Québec-Université Laval (CRCHU-UL), Québec City, QC Canada; 10 School of Health Information Science, University of Victoria, Greater Victoria, BC Canada; 11 École de santé publique, Université de Montréal, Montreal, QC Canada; 12 Gerstein Library, University of Toronto, Toronto, ON Canada

**Keywords:** Geriatrics, Health services

## Abstract

While there has been a rapid growth of digital health apps to support chronic diseases, clear standards on how to best evaluate the quality of these evolving tools are absent. This scoping review aims to synthesize the emerging field of mobile health app quality assessment by reviewing criteria used by previous studies to assess the quality of mobile apps for chronic disease management. A literature review was conducted in September 2017 for published studies that use a set of quality criteria to directly evaluate two or more patient-facing apps supporting promote chronic disease management. This resulted in 8182 citations which were reviewed by research team members, resulting in 65 articles for inclusion. An inductive coding schema to synthesize the quality criteria utilized by included articles was developed, with 40 unique quality criteria identified. Of the 43 (66%) articles that reported resources used to support criteria selection, 19 (29%) used clinical guidelines, and 10 (15%) used behavior change theory. The most commonly used criteria included the presence of user engagement or behavior change functions (97%, *n* = 63) and technical features of the app such as customizability (20%, *n* = 13, while Usability was assessed by 24 studies (36.9%). This study highlights the significant variation in quality criteria employed for the assessment of mobile health apps. Future methods for app evaluation will benefit from approaches that leverage the best evidence regarding the clinical impact and behavior change mechanisms while more directly reflecting patient needs when evaluating the quality of apps.

## Background

Over the last 10 years, mobile applications (apps) for health-related purposes have been increasingly used to support chronic disease management through mechanisms such as digital education, self-monitoring, and feedback^1–3^. While there is no single accepted definition, the World Health Organization (WHO) describes mobile health (mHealth) as the “spread of mobile technologies as well as advancements in their innovative application to address health priorities” (WHO, 2011)^4^. However, the proliferation of apps for chronic disease management poses challenges for clinicians, policymakers, and patients in understanding which apps are most likely to provide benefit. Although experimental trials remain the gold standard in determining the effectiveness of these apps, such trials are not always feasible in circumstances where the number of apps is constantly growing and their functionalities evolve over time. In place of this, researchers are increasingly turning to checklists of quality criteria that can be quickly employed to assess individual apps. The purpose of our paper is to understand this emerging mode of quality assessment for mobile apps in order to advance work on the assessment of mobile apps given their rapid proliferation in the market.

The rise of studies evaluating mobile applications using a predefined list of quality criteria raises questions about the range of criteria being used to judge their quality^5,6^. For example, in evaluating mobile applications for asthma management, Househ et al.^7^ evaluated mobile applications based on their purpose, consistency with care standards, adherence to plain language and usability guidelines, and their association between adherence to standards and price. Select studies have attempted to synthesize existing frameworks for judging the quality of digital health tools, leading to, for example, the development of the Mobile App Rating Scale (MARS)^8^. Although useful for global assessments of the appeal of the app, it often does not address the quality for a particular health-related use.

As such, building on previous reviews on quality criteria for assessing app quality^8,9^, our paper reports the results of a scoping review of the body of literature explicitly assessing mobile applications that are designed to support chronic disease management, using a predefined list of quality criteria. Such a systematic assessment of these criteria is important in order to comment on the applicability of checklist-based quality evaluations of mobile applications, and to better understand the role of such evaluations in clinical decision-making and health system planning. We analyzed the literature in order to identify the range of criteria used to assess these apps, and in the “Discussion” section elaborate on the appropriateness of those criteria in relation to the principles of evidence-based medicine and relevant principles from theories of technology use and adoption. In this way, we depart from past efforts to synthesize these criteria into an overarching framework^10^ and instead identify the challenges and opportunities of a criteria-based approach to evaluating apps intended to support self-management.

We determined that a scoping review was the most appropriate knowledge synthesis strategy, aligning with Tricco et al.’s^11^ position:

Scoping reviews are used to present a broad overview of the evidence pertaining to a topic, irrespective of study quality, and are useful when examining areas that are emerging, to clarify key concepts and identify gaps. (p. 2).

Our scoping review is motivated by the following research question: What criteria of quality are used to assess mobile applications for the support of chronic disease management in studies that review app quality?

## Results

**Fig. 1:**
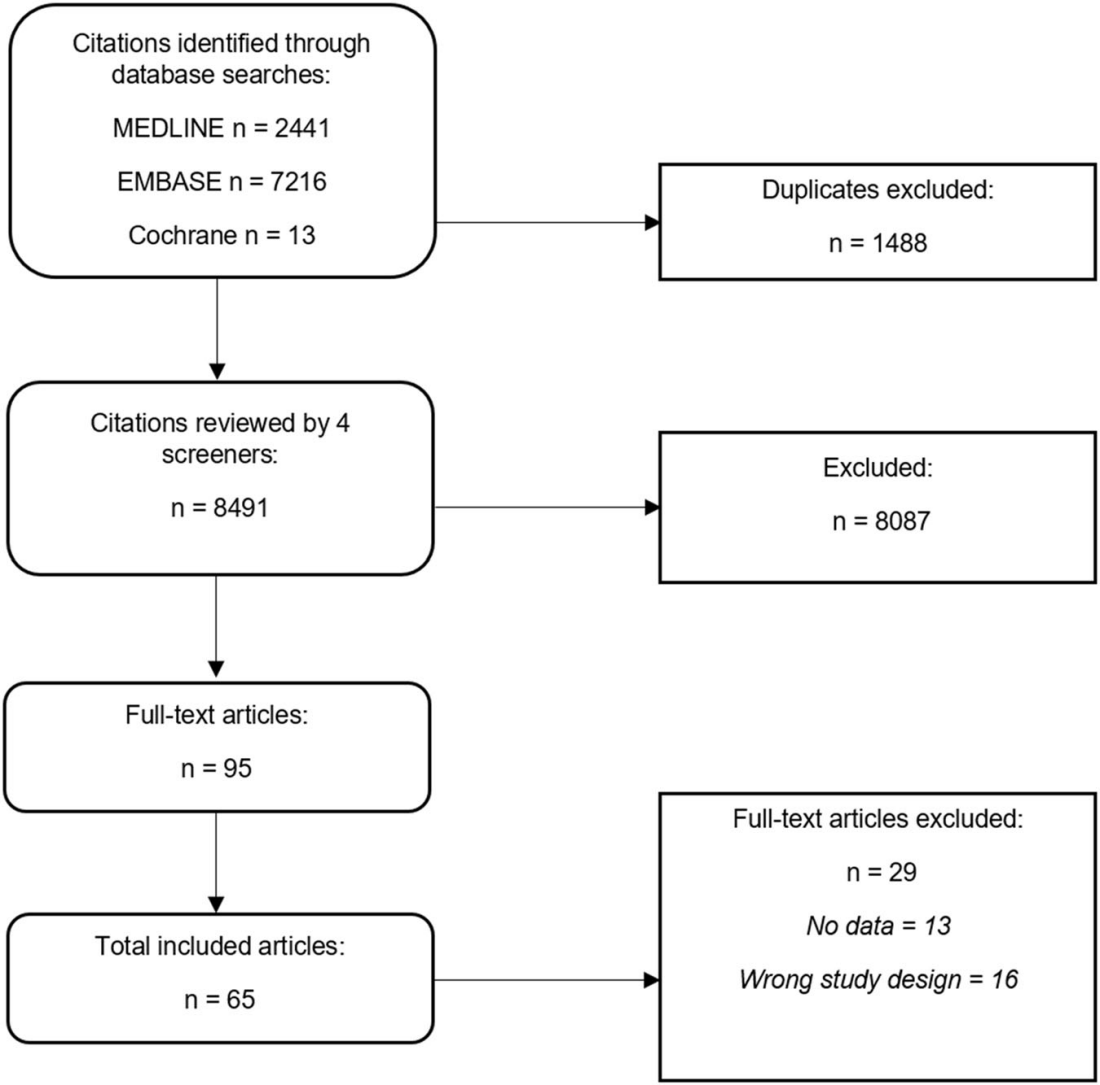
Study selection flow diagram. This figure illustrates the process by which articles were selected for inclusion in the study.

Our initial search resulted in 8491 citations once duplicates were removed. Initial review of the title and abstract excluded 8087 articles. The remaining 95 articles were reviewed by the research team resulting in 65 articles for inclusion (see Fig. 1).

Included studies were conducted between 2010 and 2017, indicating that the first relevant study included was published in 2010 despite our search frame dating back to 2001. Most explored apps related to diabetes mellitus management (24.6%, *n* = 16), weight management (15.4%, *n* = 10), mental health (10.8%, *n* = 7), and smoking cessation (10.8%, *n* = 7). Fewer explored behaviors and chronic diseases including Chronic Lung Disease (CLD), Cardiovascular Disease (CVD), eating disorders, physical activity, gout, infectious diseases, and incontinence. Reviews were conducted all over the world, with many conducted in the United States (41.5%, *n* = 27), United Kingdom (13.9%, *n* = 9), and Australia (10.8%, *n* = 7). The majority of studies were conducted in English (87.7%, *n* = 57). The reported number of apps reviewed in each study ranged from 6 to 710 (see Supplementary Table 1).

Very few studies identified a research question; however, all articles identified the objective of their review. Although there was variability in the reporting of study objectives and/or research questions, most aimed to identify and evaluate the quality of apps for a given behavior or chronic disease. See Supplementary Table 1 for a table of all 65 included articles, Supplementary Table 2 for the methods employed in each included study, and Supplementary references for complete references of all included articles.

We documented the ways in which authors established the criteria that would be used to assess the included apps, when this information was available (see Table 1). We found that in 46 cases, authors reported using a previously established framework for part or all of their assessment criteria. We define framework loosely to refer to some form of formal pre-existing guidance that directs attention toward specific attributes of the app as being most relevant for consideration. We found that authors drew primarily on three different kinds of frameworks. The first was clinical guidelines (*n* = 26), referring to published and endorsed guidance by professional groups related to the clinical management of particular conditions such as diabetes and smoking cessation guidelines. The second was behavior change guidelines (*n* = 14), informed by behavior change theory primarily coming from social psychology and implementation science, such as the Behavior Change Techniques framework. The third was technology guidelines (*n* = 14), arising from existing tools for assessing features of technology in terms of their design, uptake and use, such as the Mobile App Rating Scale. The remaining studies either used methods employed in previous studies, reviewer consensus, or the method for selecting evaluation criteria was not reported at all.

**Table 1:** Methods of criteria developments.

Name	Articles
Criteria based on specific framework	46
Criteria based on Behavior change model	14
Criteria based on Behavior change techniques	10
Criteria based on Clinical Guidelines	26
Diabetes	3
Smoking CPG	4
Criteria based on Technology Guidelines	14
Health on the Net (HON)	3
MARS	4
Silberg scale	5
Other methods to build criteria	16
PICO	1
PRISMA	1

The inductive coding process resulted in 50 unique extracted quality criteria being grouped into 6 categories (see Table 2).

**Table 2:** Quality criteria synthesis.

Name	Articles
General characteristics (*Basic descriptors of app design and usage,* *i.e. cost, date of last updated*)	42
Accuracy of App description	3
Advertising	6
App purpose	16
App release date	5
Country of origin	1
Developer or author information	14
Disclosure of external partners	9
Languages available	8
Last update	12
Number of downloads	12
Platform (iOS, Android, etc)	11
Price	25
Lite version	1
Size of App	2
Target user group	13
User ratings	19
Technical features (*Technical features related to the technology and access*)	42
Ability to customize settings	15
Availabilty of technical support	2
Internet connection requirement	6
Interoperability	22
EMR and medical programs	12
xternal devices	10
Other applications	5
Privacy and security	16
Privacy	12
Security	10
Login password process	7
Health information quality (*Whether and how the app inspires trust in the information provided*)	23
Accuracy	2
Evidence-based	12
Acknowledges areas of uncertainty	0
Clarity of purpose for information	3
Clear attribution to sources	10
Completeness of health information	3
Credibility	12
Describe quality of treatment choices	5
Refers to additional information sources	0
Unbiased information	0
Usability (*Experience of user interacting the app*)	24
Comprehensibility	3
Consistency	2
Data entry	5
Error management	3
Image Use	8
Learnability	10
Navigation	10
Efficiency	2
Intuitive controls	3
Menu navigation	3
Usefulness	4
Visual aesthetic	7
Evidence of impact (*Whether the app has empirical evaluation of its impact on outcomes*)	2
User engagement and behavior Change (*How the app attempts to engage the user and influence health behaviors*)	63
Alerts, reminders or notifications	22
Behavior change Techniques	23
Attitudes	1
Awareness	2
Help seeking	4
Hypnosis	1
Incentives	9
Intention to change	6
Knowledge	4
Recommendation or advice	16
Risks	6
Calculator	17
Community supports	36
App’s own community	5
linical team	7
Social media	12
Connection to external resources or care	8
Data export and sharing	14
Diary or journal keeping	14
Education and information provision	40
Gamification	8
Goal setting and action planning	21
Medical history profile	11
Menu planning and recipe suggestions	12
Other Engagement	46
Self-monitoring or tracking	45
Input mechanism (automated vs manual)	10
Summary charts	14

The general characteristics category included criteria that gave a basic description of the app and were used in some way by most articles. Of these criteria, those that were assessed most often were: price (*n* = 25), user ratings (*n* = 20), presence of developer or author information (*n* = 15), target user group, and platform (*n* = 13). In the category of user ratings, authors were interested both in the number of user ratings and the average rating, and few indicated whether this included written feedback. Authors described price of apps in terms of the cost of the app or whether it was free. The target audience category was grouped into a variety of sub-sets including the age and health issues of target users, and whether users were patients or healthcare professionals. Lastly, the platform was predominantly organized into availability on the Apple iOS store or Android Google Play store.

Overall technical feature criteria were often used as a measure of quality in the reviewed articles. Most represented was the ability to customize settings (*n* = 15) along with the ability to connect to EMRs and other medical devices (*n* = 13). Privacy was used as a quality criterion by 12 authors, with only 3 mentioning HIPAA compliance (no other regulatory legislation was mentioned). Only one article by Rosenfeld^12^ used multiple criteria to assess privacy. This article, focused on patients with dementia, included a detailed rubric to assess privacy among mobile apps including whether the app sold data or shared with third parties, stored an IP address, or stored cookies. The assessment was done by downloading the privacy policy. Password protection was the primary criteria by which security was assessed (*n* = 5). Only one article listed availability of technical support as a quality criterion.

The health information quality of apps were assessed by 22 articles in this study. Most often this included whether the information in the apps were developed using clinical evidence or where an explicit mention was made to theoretical or empirical evidence (n = 12). Similarly, health information quality was also judged in some studies on the basis of whether a clear attribution to a source was made for information presented in the app (*n* = 8). Additionally, credibility was assessed in 12 articles whereby authors noted the credentials of the app developers/authors (university, not for profit, etc.) or official approvals (such as those of the FDA or CE certified, as signatures of health information quality. Health information quality was also evaluated on whether the purpose was clearly communicated (*n* = 3) such as whether the information was offered in plain language, as described by Radovic^13^, and whether the purpose of information was clear (*n* = 3).

The usability of an app was assessed in 28 (*n* = 28) articles as a dimension of quality. Navigation was the most commonly reported as a dimension for assessing the usability and user experience (*n* = 10). This included evaluating menu navigation (*n* = 4), whether controls were intuitive (*n* = 3) and the level of efficiency (*n* = 2). The extent to which a system was easy to learn was also among the most commonly reported (*n* = 10) criterion. For example, some studies noted whether an app was intuitive enough to use without training. Appropriate use of images, such as simple and intuitive graphics was assessed in 8 articles. Studies described apps with strong visual aesthetic as those with minimalist designs and appropriate font sizes and graphics, a quality criterion mentioned in several records (*n* = 7). Data entry (*n* = 5), including how erroneous data is handled was also described as an indicator of quality. Other subthemes of usability and user experience included perceived usefulness, comprehensibility, and consistency.

Almost all articles (n = 63) assessed the quality of health apps by looking at the type of user engagement features included. We noted that authors tended to take one of two distinct yet overlapping approaches to assessing these features. Some authors assessed for features that explicitly advise users on strategies that are intended to help change their behavior. An example is an app suggesting changes to a person’s environment that would reduce their temptation to smoking. Others looked for features with a primary function to keep the user committed to return to the app. An example is gamification of features of the app that encourage users to advance their use. Although there are features that fall into either of these categories exclusively (changing health behavior versus promoting persistent use of the app), there are several that fall into both categories. Examples include goal-setting features, self-tracking or monitoring, and social networks, which effectively enable both functions just described.

The most frequently used methods of user engagement were self-monitoring or personal tracking (*n* = 45), followed by the provision of educational information related directly to the functions or goals of the app (*n* = 39). Also frequently used was the engagement of a wider social circle to motivate user interaction with other users or with social groups outside of the app itself (*n* = 36). This included 7 studies that looked specifically at engagement with clinical networks. The functions of goal setting or action planning (*n* = 21) and alerts, reminders or notifications (*n* = 22) were also frequently employed as criteria related to user engagement. One point to note in the user engagement category is the large number of criteria that were coded under an “other” category (*n* = 46). This category covers app content that is specific to either the disease of focus or the overarching function/goal of the app, oriented to providing novel information or functionality that seeks to engage users in ways specific to their personal circumstances.

Overall very few articles used the presence of empirical evidence of efficacy on clinical outcomes as quality criteria (*n* = 2). In one of these studies by Haskins et al, assessing for scientific evidence of effectiveness was the sole quality criteria use. Both studies conducted a systematic review of both the academic literature and commercially available app stores in order to assess for the presence of empiric evidence of efficacy.

## Discussion

Our scoping review of 65 articles that assess the quality of mobile health apps for chronic disease management shows there is minimal agreement on the methods and most appropriate criteria for this task. This is consistent with a previous review of overall methods for evaluating mobile apps, which also cited the lack of a comprehensive approach for assessing mobile health app quality^14^. In most studies we reviewed, assessments were done with “boutique” criteria developed for particular instances of apps, as opposed to the use of pre-determined or standardized list. There was also significant variation in the number of apps reviewed, with several articles reporting reviews of over 700 apps (*n* = 7), while 15 articles reviewed less than 20 apps. Overall, studies used over 50 distinct quality criteria, with 23 giving no insights into how criteria were selected. Although 43 studies attempted to use existing frameworks in the development of quality criteria, there was significant variation in the body of literature from which they drew (including clinical practice guidelines, technical guidelines for websites, and behavior change frameworks). Further, no single framework of quality criteria used by authors in our review actually includes all 6 dimensions of quality we identified. For example, each of the studies that employed the Mobile App Rating Scale (MARS) added additional features of quality in an effort to provide a more comprehensive assessment.

Given the breadth of apps and evaluation criteria identified in our scoping review, we suggest that a hybrid approach between consistent criteria for more technical consideration (e.g., privacy) and ‘boutique’ features of each app (e.g., relating to topic-specific evidence-based guidelines) may be more appropriate than a single universal checklist. However, it is evident more research is needed to establish a more consistent, credible approach to identifying the criteria that are most likely to represent overall app quality and effectiveness.

Based on our analysis, we propose 3 overarching goals to guide future attempts to build a set of quality criteria for app development: (1) quality criteria should be carefully selected to prioritize existing evidence and knowledge over ease of assessment, (2) behavior change theory, particularly growing knowledge relating specifically to the potential of digital health apps to influence behavior^14–16^, should be central to the evaluation of these apps, and (3) criteria should explicitly incorporate the patients view, in order to be situated more clearly in the goals and needs of patients living with chronic diseases.

A review of the synthesized quality criteria suggests that current studies may be selecting quality criteria based in part by the ease of evaluation and not its ability to act as a proxy for quality and effectiveness. For example, most studies (*n* = 42) used quality criteria that measured general characteristics that could be collected without downloading or using the app itself. User ratings were one of the most commonly used criteria, but studies show that there is a limited correlation between ratings and objective measures of effectiveness^17,18^. Similarly, a number of downloads and size of app are unlikely to be direct predictors of app quality^18^. One possible exception is the date of the last update; several groups have flagged this as a possible marker for high-quality apps^18^.

While it is well known that the design and usability of an app can be the determining factor in if and how people use an app^19,20^, fewer than half of studies employed usability criteria in their quality assessment. This is particularly surprising given there is a significant body of literature that gives evidence-based recommendations for improving the usability of digital interfaces. For example, Nielson’s 10 Usability Heuristics for User Interface Design highlights the importance of aesthetic and minimalist design that helps users recognize, diagnose, and recover from errors, flexibility, and efficiency of use, and promotes error prevention^21^. While several studies included criteria aligned with these principles, we suggest that all assessments of quality should include measures that reflect the usability of the app.

Further, the infrequent use of empirical evidence of effectiveness (n = 2) as a quality criteria arises as a clear gap in current quality assessment. There have been a growing number of empirical studies looking at clinical effectiveness of apps. The presence of high quality, emperic evidence of clinical impact should be a quality heurisitic for all future app quality reviews. If an app in question has not been studied directly, emeperic evidence of similar apps should still be considered in quality assessements. For example, a review of randomized-control trials suggests all diabetes apps should include education on how to prevent complications of the disease to maximize clinical impact on HbA1c reduction^22^. Studies in our review more often used developer credibility and health information quality as a proxy for clinical validity. While the health information quality criteria were most often derived from the HON code^23^ and Silberg Scale^24^, these frameworks were initially designed for health websites, and their effectiveness for assessing the clinical validity of digital health apps has not been established.

Given our review included studies that were focused on apps for chronic disease management, a significant focus on mechanisms to change health behaviors was expected. Evaluations generally assessed quality around behavior change based solely on the presence of user engagement features. Very few studies relied on established models or theories of behavior change to develop quality criteria for mobile apps (i.e. only 14 studies used a behavior change framework to develop its quality criteria). These results are consistent with those by McKay et al.^14^ who found only 4 out of 38 studies evaluating apps included a behavior change framework. We expected that health promotion and behavior change frameworks, such as Michie’s behavior change wheel^25^, would be more frequently incorporated into ratings of quality for apps designed to promote chronic disease management. However, this was not the case in our findings.

While the thoughtful use of user engagement features may drive app usage and overall impact on behavior change^26^ it is unclear if more is always better as most reviewed studies assumed^27^. It is possible that careless or overuse of these features could lead to user overload, fatigue, or worse health outcomes^28,29^. Further, given growing evidence that digital tools are most effective in changing health behaviors when integrated into a person’s clinical team, it seems appropriate that this be more prominent criteria of app evaluations^15,30,31^.

Our results bring to question whether current quality criteria for apps to better manage chronic disease management align with the known needs of patients. Typically, while most patients express significant interest in using mobile health apps, they stop using them within months of initiation^32^. This suggests that current apps are not sufficiently addressing patient expectations or needs regarding their health. High burden of data entry was often cited as a reason for disliking or abandoning an app^17,33^, yet as discussed above, most included studies assumed apps with more user engagement features were better quality. More broadly, a review of patient-centered chronic disease management highlighted the importance of “legitimizing the illness experience” and “offering realistic hope”^34^. Based on our review, no evaluations of app quality included such patient-centered criteria in their assessment. Further, it is unclear that assessing quality through academic papers alone is sufficient to meet patient needs, as most patients do not access these articles. Greater efforts are needed to ensure patients and care providers have access to these quality insights when making care decisions through accessible methods of knowledge translation such as websites or clinician education modules.

While patients, along with healthcare providers and regulatory bodies, frequently cite privacy and security as an essential component of digital health evaluations^33,35,36^ only 16 articles used these quality criterion. However, we suggest that clear assessments of apps for privacy and security that are aligned with guidance from regulatory organizations^37,38^ are imperative to ensure patient’s needs are being met. A review of health apps in the European Union found many do not follow regulatory guidelines around app privacy and security^39^. Further, serious lapses in the security of recommended digital health apps shut down a large “app store” by the credible National Health Services (NHS)^40^. This reality can impact how patients use health apps, with health professionals frequently citing privacy and security concerns as a limiting factor in their use of health apps with patients^14,41^. It is possible that the lack of analysis around privacy and security is due to the limited knowledge of study authors regarding the details of digital health privacy and security. Again, it seems likely that quality criteria were selected for ease of evaluation as opposed to importance to end users (i.e. security).

### Limitations

Our study has two potential limitations that should be understood by readers. First, we employed a method for screening potentially included studies wherein 8 research staff were involved in making article inclusion/exclusion decisions. We ensured that each staff member was trained appropriately, and that each record was screened by two individuals independently, in order to account for the large number of reviewers. This was done to enhance the feasibility of the review. Second, literature assessing the quality of mobile apps for health is a rapidly evolving field. It is possible that the field will have developed substantially by the time this article is published. However, we believe it remains extremely important to leverage in-depth analyses of the criteria to evaluate mobile apps for health, as work such as this lays a foundation for the development and assessment of such apps in the future. More recent articles reviewing the quality of apps continue to show significant variability in the criteria used and their alignement to patient needs^42–46^.

## Conclusions

This scoping review reported on a large number of studies that focused on assessing the quality of mobile health apps for chronic disease management. There continues to be significant variation in quality criteria employed for assessment, with no clear method for developing the included criteria. Currently no single existing framework addressed all 6 identified dimensions of mobile health app quality. Future methods for app evaluation will benefit from a clearer approach to quality assessments that balance the needs for standardized quality criteria with the unique needs of different types of health apps. Research is already emerging that is moving toward this approach to assessing app quality.^47^ Future work should focus on understanding how to leverage best evidence to evaluate apps across a broad range of criteria, while working to understand how to best impact patients and care providers decision making around using health apps.

## Methods

We used a scoping review methodology to review a body of literature that is quickly emerging, drawing on methodological guidance offered by Tricco et al. (2016) and Levac et al. (2010)^48^. We refined our research question by selecting exemplar papers^7,49–53^ and discussing their contents as a group. An academic librarian (EB) constructed the search parameters and refined the search string over several iterations to ensure we had a comprehensive search that included all exemplar papers (search details available in Supplementary Table 3). The final list of chronic diseases was informed by Hamine et al. (2015) based on their high global burden and includes diabetes mellitus (DM), cardiovascular diseases (CVDs), and chronic lung diseases (CLDs)^1^. Additionally, we chose to include search terms related to mental health, given the growing prevalence of mental health apps in this body of literature. In addition to specific chronic diseases, our search also included health behaviors that are widely recognized as supporting the management of multiple chronic disease including medication adherence, weight loss/management, smoking cessation, alcohol consumption, and substance use^54^.

### Search strategy

We ran searches in three electronic databases through September 2017. The search databases include MEDLINE, EMBASE, and the Cochrane Central Database of Controlled Trials. We did not hand search gray literature because our primary focus was the ways in which our topic has been addressed in academic literature. The searches were stored on the Endnote software. Medical subject heading (MeSH) and selected keywords were searched within three categories of concepts (mobile applications, chronic disease, and behavior change), as per the detailed search strategy available in Supplementary Table 3. We restricted the searches to only yield studies from 2000 onwards to capture the rise of mobile phone use. We only retrieved titles and abstracts in English. All search results were filtered to include only those references that include the word “review” in the reference information.

### Inclusion and exclusion criteria

Our inclusion criteria included: published studies that use a set of quality criteria to directly evaluate two or more mobile applications intended to promote chronic disease management through patient-facing apps. Our exclusion criteria included: prospective studies involving primary data collection regarding the use of an app or its actual clinical effects, and those focused on apps for diagnostic purposes or acute care management. Conference proceedings were excluded.

Titles and abstracts were reviewed for inclusion by a team of eight research staff working concurrently in pairs to review the titles and abstracts retrieved during the search. All titles and abstracts were independently reviewed by two members of the research team. The two scientific leads of the project (a Scientist JS and Clinician Scientist PA) trained the research staff regarding the objectives of the study and the process of title and abstract review. Each of the research staff completed a training sample of screening 100 titles and abstracts, and decisions related to inclusion and exclusion on those 100 titles and abstracts were then discussed together as a group. Where research staff was unsure about inclusion or exclusion, or where there was a conflict between the decisions made by the two independent staff members, our team resolved disagreements by discussing until we reached a group agreement.

### Data extraction and coding

The full text of included articles were downloaded and saved to a local computer, and basic descriptive details were extracted for reporting. These details included the methods employed in studies to select apps, descriptive information about reviewers, review processes followed, and the methods by which the criteria employed in each study were developed (where this information was provided).

Because a very wide variety of criteria were applied to assess the quality of apps in the included studies, the research team developed a strategy to group these criteria in a meaningful way that could inform the generation of a codebook to be applied to all included studies. The team read a sub-set of 30 papers in order to generate the codebook, which was then applied to the remainder of the studies and modified as new codes were identified. Inductively identified codes were generated to represent the quality criteria applied in each study. Four members of the research team then got together to refine the inductively generated list of criteria and group them thematically. The thematic groupings of quality criteria formed the foundation for the analysis of the scoping review. All included articles were then coded on NVivo using the codebook. The team continued to meet through the coding process to review results and iterate on the developed coding schema. A second phase of analysis determined which criteria were used most frequently and generated discussion points with the research team about (a) which criteria might be missing, and (b) which criteria are most essential for a pragmatic app evaluation too. These points are revisited in the “Discussion” section of this paper.

## Supplementary information

Supplementary Information

## Data Availability

The authors declare that all data supporting the findings of this study are available within the paper and its supplementary information files.
