# Supplementary material for: Assessing the quality of mobile applications in chronic disease management: a scoping review
Source: NPJ Digit Med. 2021 Mar 10;4:46. doi: 10.1038/s41746-021-00410-x (PMC7946941; doi:10.1038/s41746-021-00410-x)
Supplement: Supplementary file 1 — Supplementary Information [file 41746_2021_410_MOESM1_ESM.pdf]

Supplementary Table 1 - Characteristics of Included Studies

Article #	Authors	Year	Country	Language	Database searched	Date range	Final number analyzed	Search terms (content)
1	Lorien C. Abroms, J. Lee Westmaas, Jeuneviette Bontemps-Jones, Rathna Ramani, Jenelle Mellerson	2013	United States	English	Apple and Google Play	February 2012	98	quit smoking, stop smoking, and smoking cessation
2	Lorien C. Abroms, Nalini Padmanabhan, Lalida Thaweethai, Todd Phillips	2011	United States	English	Apple	June 2009	47	quit smoking, stop smoking, and smoking cessation
3	Aroub A. Alnasser, Raja E. Amalraj, Arjuna Sathiaseelan, Abdulrahman S. Al-Khalifa, Debbi Marais	2016	Saudi Arabia	English	Apple, Google Play, Blackberry, Nokia Ovi, Samsung, and Windows Phone	March 2013	65	Arabic translations of the words “weight” and “diet” or “regimen.”
4	Dari Alhuwail	2016	Kuwait	Arabic	Apple and Google Play	September to December 2015	18	Sukkar- ;سكر; Alsukkar- السكر ; Sukkari- سكري ; Alsukkari- السكري
5	Madlen Arnhold, Dipl-Soz, Mandy Quade, and Wilhelm Kirch	2014	Germany	English and German	Apple and Google Play	April 2013	656	Diabetes, Blood Sugar/Blutzucker, Glucose/Glukose
6	Andrea Basilico, Sara Marceglia, Stefano Bonacina, Francesco Pinciroli	2016	United States (authors from Italy)	English	Apple	April 1, 2014	41	Diabetes

7	Marco Bardus , Samantha B. van Beurden, Jane R. Smith and Charles Abraham	2016	United States	English	mHealthApps repository (includes Apple and Google play apps)	April 2015	23	Health and Fitness category
8	Jessica Y. Breland, Vivian M. Yeh, Jessica Yu	2013	United States	English	Apple	March 17, 2012	23	diabetes
9	Maria Rosa Cantudo Cuenca, Maria Dolores Cantudo Cuenca, Ramon Morillo Verdugo	2013	Spain	English	Apple app and Google Play	May 2013	41	HIV', 'AIDS' and 'acquired immune deficiency syndrome'
10	Petra Povalej Brzan, Eva Rotman, Majda Pajnkihar, Petra Klanjsek	2016	Slovenia	English	Apple, Google Play, and Windows Phone	November 12, 2015 to December 30, 2015	9	Diabetes
11	Jounghwa Choi, Ghee-Young Noh, Dong-Jin Park	2014	South Korea	Korean and English	Apple and Google Play	November 2013	175	"smoking" and "smoking cessation," either in Korean or in English
12	Thomas Carter, Stephen O'Neill, Neil Johns, Richard R.W. Brady	2013	United Kingdom	English	Apple, Google Play, Windows Phone, Blackberry, Nokia and Samsung	March 13, 2012	49	Peripheral artery (arterial) disease, varicose veins, aortic aneurysm, carotid artery disease, amputation, ulcers, hyperhidrosis, thoracic outlet syndrome, vascular malformation, and lymphatic disorders.
13	Taridzo Chomutare, Luis Fernandez-Luque, Eirik Årsand, Gunnar Hartvigsen	2011	Norway	English	Apple, Google Play, Windows Phone, Blackberry, Nokia Symbian	February 2011	137 (82 installed)	Diabetes OR glucose
14	David Crane, Claire Garnett, James Brown, Robert West, Susan Michie	2015	United Kingdom	English	Apple and Google Play	April to May 2014	61	"alcohol" and "drink"
15	Cohn A, Hunter-Reel D, Hagman B, Mitchell J	2010	United States	English	Apple	Not specified	767	alcohol, drinking, recovery, abstinence, harm reduction, moderation, blood alcohol, alcoholics anonymous, self-help, 12-step, recovery, and cognitive therapy

16	Lindsey Dayer, Seth Heldenbrand, Paul Anderson, Paul O. Gubbins, and Bradley C. Martin	2013	United States	English	Apple and Google Play	August to September 2012	10	Adherence, compliance, dose, drug, med(s), medication(s), remind, reminder, Rx, take, therapy, treat, and treatment.
17	Alaina Darby, Matthew W. Strum, Erin Holmes, and Justin Gatwood	2016	United States	English	Apple, Google Play, Windows Phone	Not specified	42	Diet, eating, food, food diary (or journal or tracker), health, nutrition, nutrition diary (or journal or logging), and nutritional tracking
18	Andrew P Demidowich, Kevin Lu, Ronald Tamler, Zachary Bloomgarden	2012	United States	English	Google Play	April 2011	42	glucose, diabetes, insulin, a1c and blood sugar
19	Andjela Drincic, Priya Prahalad, Deborah Greenwood, and David C. Klonoff	2016	United States	English	Apple and Google Play	March 10, 2016	14	Not explicitly indicated but the authors included apps intended to support blood glucose (BG) monitoring and DM self-management in patients with type 1 DM (T1DM) and type 2 DM (T2DM).
20	Divyanshu Dubey, Amod Amritphale, Anshudha Sawhney, Nupur Amritphale, Pradeep Dubey, and Ambarish Pandeya	2014	United States	English	Apple and Google Play	July 27, 2013	93	stroke, brain attack, intracranial hemorrhage, subarachnoid hemorrhage, cerebral infarction
21	El-Gayar O., Timsina P., Nawar N., Eid W.	2013	United States	English	Apple	August 2012	71 apps; 16 articles	diabetes
22	Fairburn, C., Rothwell, E.	2015	United Kingdom	English	Apple, Google Play, Blackberry, Nokia, Windows Phone, Amazon App Store and Google	Up to and including July 31st, 2014	44	eating disorder, eating disorders, anorexia, anorexia nervosa, bulimia, bulimia nervosa, binge, and binge eating
23	Formagini T., Ervilha, R., Machado, N., de Andrade, B., Gomide, H., Ronzani, T.	2017	Brazil	Portuguese	Apple and Google Play	April 2015	15	“how to stop smoking”, “quit smoking”, “stop smoking”, “smoking”, and “cigarette”
24	Franco R., Fallaize R., Lovegrove J., Hwang F.	2016	United Kingdom	English	Google Play and iTunes App store	November 2015	13	Calorie(s), diet, diet tracker, dietician, dietitian, eating, fit, fitness, food, food diary, food tracker, health, lose weight, nutrition, nutritionist, weight, weight loss, weight management, weight watcher, and ww calculator

25	Gao C., Zhou L., Liu Z., Wang H., Bowers B.	2017	China	English (inclusion criteria specified only apps that had a Chinese-language user interface)	Apple and the 360 mobile Assistant	September 2015	71	Diabetes mellitus and blood sugar
26	Haase J, Dorsch, M.	2017	United States	English	Apple	September 5, 2014	30	“medication adherence” and “medication reminder”
27	Hale, K., Capra, S., and Bauer, J.	2015	Australia	English	Apple and Google Play	March 2014	14	"Top 200 Free", "Top 200 Grossing", "diabetes", "GI" (glycemic index), "glycemic index," "relaxation," "confidence," and "CBT" (cognitive behavioral therapy).
28	Hales, S., Dunn, C., Wilcox S., Turner-McGrievy, G.	2016	United States	English	Apple and Google Play	September 30, 2015	29	"weight loss and photo”, “diet and photo”, “food and photo”, “weight loss and picture”, “diet and picture”, “food and picture”
29	Haskins, B., Lesperance, D., Gibbons, P., Boudreaux, E.	2017	United States	English	Apple, Blackberry, Google Play, and Windows Phone	October 2015	6	"quit smoking”, “stop smoking”, and “smoking cessation”
30	Heldenbrand, S., Martin, B., Gubbins, P., Hadden, K., Renna, C., Shilling R., and Dayer L.	2015	United States	English	Apple, Google Play, and Blackberry	June 2014	367	Adherence, compliance, dosage, dose, drug, med(s), medication(s), pharm, pharmacy, pill(s), prescription, remind, reminder, Rx, script, take, therapy, treat, and treatment. In each online app store, the “Health and Fitness” and “Medical” categories were also comprehensively searched
31	Hoeppner, B., Hoeppner, S., Seaboyer, L., Schick, M., Wu, G., Bergman, B., and Kelly, J.	2015	United States	English	Google Play	October 1, 2013 to May 31, 2014	225	“smoking,” “smoking cessation,” “quit,” “stop smoking,” and “quit smoking”

32	Hoppe, C., Cade, J., and Carter, M.	2017	United Kingdom	English	Google Play	October 27, 2014 to June 9, 2015	40	‘diabetes’, ‘diabetes type 1’, ‘diabetes type 2’, ‘gestational diabetes’
33	Househ, M., Hossain, N., Jamal, A., Zakaria, N., Elmetwally, A., Alsalamah, M., Khalifa, M.	2017	Saudi Arabia	English	Google Play	October 26, 2014	15	“Asthma,” “Lung Function” and “Peak Flow”
34	Huckvale, K., Car, M., Morrison, C., and Car, J.	2012	United Kingdom	English	Google Play, Apple, Blackberry and Windows Phone	August 2011	103	asthma, lung function, peak flow and inhaler.
35	Huckvale, K., Adomaviciute, S., Prieto, J., Khee-Shing Leow, M., Car, J.	2015	United Kingdom	English	Apple and Google Play	August 2013	46	insulin', 'glucose', 'bolus', 'diabetes', 'diabetic', 'DM', 'BG', 'dose', 'dosing', 'calculator', 'calc', 'algorithm'
36	Huguet, A., Rao, S., McGrath, P., Wozney, L., Wheaton, M., Conrod J., and Rozario, S.	2016	Australia	English	Apple and Google Play	November 2027	117	Depression
37	Issom, D-Z., Woldaregay, A., Chomutare, T., Bradway, M., Arsand, E., and Hartvigsen, G.	2015	Norway	English	Google Play, Blackberry, Apple, Nokia Ovi and Windows Phone	March to April 2015	53	‘diabetes’ AND ‘mobile’ OR ‘app’ OR ‘self-management’
38	Jacobs, M., Cobb, C., Abroms, L., and Graham, A.	2014	United States	English	Facebook	August 2013	9	“smoking”, “quit”, “cessation”, “cigarette cessation” in the Facebook general search toolbar with “Apps/Games”
39	Jeon, E., Park, H., Min, Y., Kim, H.	2014	Korea	Korean	Apple	September 23 and September 27, 2013	104	'obesity + management,' 'weight + management,' 'weight + loss,' 'weight + exercise,' 'weight + diet,' 'weight + calories,' and 'diet,'
40	Juarascio, A., Manasse S., Goldstein, S., Forman, E., and Butryn, M.	2015	United States	English	Apple and Google Play	Not specified	6	‘Eating disorders,’ ‘Anorexia,’ ‘Bulimia,’ ‘Binge eating,’ and ‘Binge Eating Disorder.’
41	Kumar, N., Khunger, M., Gupta, A., and Garg, N.	2015	United States	English	Apple and Google Play	May 2014	107	"hypertension" and "high"
42	Kumar S., Mehrotra S.	2017	India	English	Google Play	October to November 2016	33	Depression

43	Larsen, M.E., Nicholas, J., Christensen, H.	2016	Australia	English	Apple and Google Play	Not specified	49	Suicid*; parasuicid*; kill me/myself/ yourself; take my/your [own]life; self[–]harm; DSH
44	Martínez-Pérez B., de la Torre-Díez I., López-Coronado M., Herreros-González J., López-Coronado, M., and Herreros-González, J.	2013	Spain	English	Apple and Google Play	May 2013	710	infarction, heart attack, heart failure, heart disease, fibrillation, coronary heart, angina, and arrhythmia
45	Martínez-Pérez, B., de la Torre-Díez, I., and López-Coronado, M.	2013	Spain	English	Apple, Google Play, BlackBerry, Windows Phone, and Nokia Ovi	April 2013	8	hearing loss and iron-deficiency anemia, hearing loss, migraine, low vision, asthma, diabetes mellitus, osteoarthritis (OA), and unipolar depressive disorders
46	Modave, F., Bian, J., Leavitt, T., Bromwell, J., Harris C., and Vincent, H.	2015	United States	English	Apple	April 6, 2015	30	"workout" and "training" and restricted search to the "health and fitness" category
47	Moodley, A., Mangino, J.E., and Goff, D.A.	2013	United States	English	Apple and Google Play	Apple: December 1, 2012 to January 8, 2013<br>Google: April 2013	24	"infection", "infectious diseases", "pediatric", "infection control", "immunizations", "vaccines", "antimicrobials", "antibiotic", "pneumonia", "CDC", and "influenza" Second search in apple: "reference", "imaging", "personal care", "medical education", "patient education", and "EMR & patient monitoring"; Google: "infectious diseases"
48	Morrissey E.C., Corbett T.K., Walsh J.C., Molloy G.J.	2016	Ireland	English	Apple and Google Play	February 2015	166	Medication, pill, adherence, compliance, monitor, remind, tracker, diary and management
49	Myint M., Adam A., Herath S., and Smith G.	2015	Australia	English	Apple, Google Play, and Blackberry	August 21, 2014	24	Bladder, bedwetting, bladder diary, enuresis, incontinence, and wetting
50	Nguyen, E., Bugno, L., Kandah, C., Plevinsky, J., Poulopoulos, N., Wojtowicz, A., Schneider, K.L. and Greenley, R.N.	2016	United States	English	Apple	May to June 2015	101	''adherence,'' ''compliance,'' ''medication reminder,'' ''prescription reminder,'' ''pill reminder,'' and ''medicine reminder.'

51	Nguyen, A.D., Baysaria, M.T., Kannangara D., Tariq, A., .Lau, A., Westbrook, J.I., and Day, R.O.	2016	Australia	English	Apple and Google Play	May 2015	6	Gout
52	Nicholas, J., Larsen, M.E., Proudfoot, J., and Christensen, H.	2015	Australia	English	Apple and Google Play	July 16, 2014	82	bipolar, bi-polar, “manic depression”, “mood swings”, “mania” AND “mood”, cyclothymia, and cyclothymic.
53	Nikolau, C.K., and Lean, M.E.	2017	Belgium and Scotland (apps from all over the world)	English	Apple, Google Play, Amazon, Windows Phone, and Blackberry	January to February 2016	500	‘weight’, ‘calorie’, ‘weight-loss’, ‘slimming’, ‘diet’, ‘dietitian’ and ‘overweight’.
54	Pagoto, S., Schneider, K., Jojic, M., DeBiasse, M., and Mann, D.	2013	United States	English	Apple and Google Play	Not specified	30	Not specified
55	Patel, R., Sulzberger, L., Li, G., Mair, J, Morley, H., Ng-Wai Shing, M., O’Leary, C., Prakash, A., Robilliard, N., Rutherford, M., Sharpe, C., Shie, C., Sritharan, L., Turnbull, J., Whyte, I., Yu, H., Cleghorn, C., Leung, W., and Wilson, N/	2015	New Zealand	English	Apple	Not specified	120	Not specified
56	Penzenstadler, L., Chatton, A., Van Singer, M., and Khazaal, Y.	2016	Switzerland	English	Apple	July to August 2014	137	‘alcohol’, ‘alcohol addiction’, ‘alcohol help’ and ‘stop drinking’.
57	Radovic, A., Vona, P.L., Santostefano, A.M., Ciaravino, S., Miller, E., and Stein, B.D.	2016	United States	English	Apple and Google Play	March 2014	208	mental health, depression, anxiety, schizophrenia, bipolar, trauma, trauma in schools, post traumatic stress disorder (PTSD), child trauma, and bullying
58	Rosenfeld, L., Torous, J., and Vahia, I.V.	2017	United States	English	Apple	April 20, 2016 to May 1, 2016	33	Medical + dementia AND health & fitness + dementia

59	Rossi, M.G., and Bigi, S.	2017	Italy	English and Italian	Apple and Google Play	October 2015	17	“diabete” (diabetes), “diabete glicemia” (diabetes glycemia), “diabete educazione” (diabetes education), “diabete sport” (diabetes sport), “diabete movimento” (diabetes exercise), “diabete dieta” (diabetes diet), “diabete alimentazione” (diabetes nutrition)
60	Santo, K., Richtering, S.S., Chalmers, J., Thiagalingam, A., Chow, C.K., and Redfern, J.	2016	Australia	English	Apple and Google Play	Apps updated in 2015	10	medication reminder, medication pill reminder, pill reminder, meds reminder, medication tracker, medication management, Rx, and medication.
61	Sobnath, D.D., Philip, N., Kayyali, R., Nabhani-Gebara, S., Pierscionek, B., Vaes, A.W., Spruit M.A., Kaimakamis, E.	2017	United Kingdom	English	Apple, Google Play, BlackBerry, Windows Phone, and Nokia Ovi	Not specified	20	"COPD” and “COPD Management.”
62	Stevens, D.J., Jackson, J.A., Howes, N., and Morgan, J.	2013	United Kingdom	English	Apple, Google Play, and Blackberry	December 2, 2012	28	Bariatric, obesity, weight loss, gastric band, gastric sleeve, gastric balloon, gastric bypass, roux-en-y
63	Van Singer, M., Chatton, A., and Khazaal, Y.	2015	Switzerland	English	Google Play	February to March 2014	52	“stop panic,” “stop panic attack,” “panic attack,” “PD,” “anxiety attacks,” and “anxiety disorder.”
64	Xiao, Q., Lu, S., Wang, Y., Sun, L., and Wu, Y.	2017	China	English and Chinese	Apple, Google Play, 360 app market, Wan Dou Jia, Baidu mobile, and Yin Yong Bao	September 2015	151	“heart,’’ ‘‘heart disease,’’ ‘‘coronary heart disease,’’ ‘‘coronary disease,’’ ‘‘coronary artery disease,’’ ‘‘ischemic heart disease,’’ ‘‘acute coronary syndrome,’’ ‘‘coronary atherosclerosis,’’ ‘‘angina,’’ ‘‘angina pectoris,’’ ‘‘myocardial infarction,’’ ‘‘hypertension,’’ ‘‘blood pressure,’’ ‘‘high blood pressure,’’ ‘‘arrhythmia,’’ ‘‘valvular heart disease,’’ ‘‘atrial fibrillation,’’ ‘‘heart failure,’’ ‘‘heart attack,’’ and ‘‘cardiac dysfunction’’ written either in Chinese or English
65	Zhang, M.W., Ho, R.C., Hawa, R., Sockalingam, S.	2015	North America	English	Apple and Google Play	June 2014	39	Bariatric surgery, weight loss surgery, gastric band, gastric sleeve, and gastric bypass

Supplementary Table 2 - Included Articles Methods

Article #	Method to identify final sample	Brief description of review process (method)	No. of Reviewers	Reviewer skillsets	Analyses Performed
1	Limit[ed] the analysis to the top 50 most popular English-language apps for each operating system.	Each item was coded as 0 indicating “not present at all,” 1 indicating “partially present,” or 2 indicating “fully present.” The maximum possible score on the index for an app was 42 points. If coding scores differed by 1 point, the two scores were averaged.	2	Not specified; Authors: From the George Washington University (Abroms, Ramani), Washington, District of Columbia, and the American Cancer Society (Westmaas, Bontemps-Jones, Mellerson), Atlanta, Georgia	Statistical comparisons of means were conducted across operating systems using t-tests. Tests of associations between Index Scores and popularity were conducted with Pearson correlations.
2	Of 62 apps [that were initially identified], 10 were excluded because their descriptions in the iTunes store indicated they were irrelevant for reducing or quiting smoking...; four were eventually removed from the sample because they were no longer in the iTunes store at the time of downloading; one app was removed because the basic and deluxe versions proved to be identical.	Each app was independently coded by 2 reviewerss for its (1) approach to smoking cessation and (2) adherence to the US Public Health Service’s 2008 Clincial Practice Guidelines for Treating Tobacco Use and Dependence. Each app was also coded for its (3) frequency of downloads.	2	Not specified; Authors: From the Department of Prevention and Community Health, George Washington University School of Public Health and Health Services (Abroms, Thaweethai); the Academy for Educational Development (Phillips), Washington DC; and the Division of Cancer Control and Population Sciences, National Cancer Institute (Padmanabhan), Rockville, Maryland	Each app was independently coded by two reviewers on each of the 20 guidelines using a scale that ranged from 0 to 3. A 3 indicated that the feature was fully present, and a 0 indicated that it was not present at all.

3	When an app had the same name or was developed by the same company as another app that was already included in the study, it was considered to be a duplicate content and excluded. Additionally, we excluded apps that were duplicated in the search results of the various search terms used. Following a review of both free and paid versions of apps, we found that the free version (where a paid version was also available, n=18) only provided a portion of the functionality or advice available in the paid version. For this reason, apps that included both free and paid versions were counted as one app, and only the paid version was downloaded.	Prior to downloading, we reviewed the description pages of all apps... The description page was used as an initial screening tool to determine whether weight loss was the stated purpose of the app. We downloaded all apps in the Arabic language that had weight control content to include in the study, and we systematically explored their functionalities over one period of use.	1*<br><br>*Lead author downloaded and tested apps under the supervision of the research team; uncertainties were discussed by the research team; and an independent rater reviewed a random selection of the studies (21.5%) to ensure high interrelater reliability	Lead author (main reviewer): Department of Food Science and Nutrition, King Saud University, Riyadh, Saudi Arabia. Research team: (1)Department of Food Science and Nutrition, King Saud University, Riyadh, Saudi Arabia; (2) Division of Applied Health Sciences University of Aberdeen, Aberdeen, UK; (3) Computer Laboratory, University of Cambridge, Cambridge, UK	If the app was capable of performing any of the following tasks, they were allocated one point per task, up to a maximum of 13 points: (1) determining and explaining BMI, (2) recommending and tracking daily servings of fruit and vegetables, (3) recommending daily physical activity, (4) advising the user to drink water instead of soda or juice and tracking their daily intake of water, (5) allowing for the recording of daily food intake, (6) providing a calorie tracker to maintain calorie balance, (7) providing weight-loss goals of 1–2 lb/week, (8) providing information about portion control, (9) recommending that the user read and understand nutrition labels, (10) providing a way to track weight, (11) providing a way to keep a physical activity journal, (12) offering suggestions for meal planning, and (13) offering a private social network or the capability of being linked to popular social media such as Facebook, Twitter, or Instagram for social support.<br><br>Descriptive statistics were used to summarize the total number and percentages of evidence-informed practices included in the Arabic apps. The median and interquartile range (IQR) were calculated for nonnormally distributed data, such as stars representing user opinions (ranging from 0.5 to 5 stars), and number of user ratings (number of users who gave a star rating on the app

					description page)...We used latent class analysis to identify whether there were distinct classes or subgroups within the apps included in the study. No prior prediction of the outcome was made. The number of classes was determined by fit indices [14, 15], such as Bayesian information criteria (BIC), adjusted Bayesian information criteria (ABIC), Akaike's information criteria (AIC), and entropy and interpretability or model usefulness. The number of classes that minimized AIC and BIC was chosen from the modeling. The profile or description of these classes was based on the response pattern of evidenceinformed practice features for that class. Based on the posterior probability of class membership, each app was categorized into one of the classes. The app price was categorized into "paid" and "free". Fisher's exact test was used to investigate associations between pricing and class, based on the posterior probability...Apps that did not have any stars or ratings were excluded from the comparison, and the median stars and ratings of the two classes were compared using the Mann–Whitney test. A p value of less than 0.05 was considered significant.

4	Only apps catering to the Arabic language speakers, or apps that have an Arabic interface were considered.	Detailed information on each app were extracted and reviewed including description, functionality, and price. All free apps were downloaded and their functions were examined closely.	Unclear<br><br>(assumption: 1 as there is 1 author)	Not specified; Author from: College of Computer Science & Engineering, Kuwait University	Thematic
5	Every hit was reviewed in terms of its relevance and explicit link to diabetes mellitus.	We identified relevant keywords, comparative categories, and their specifications. Subsequently, we performed the app review based on the information given in the Google Play Store, the Apple App Store, and the apps themselves. In addition, we carried out an expert-based usability evaluation based on a representative 10% sample of diabetes apps.	Not specified<br><br>(usability evaluation was performed by three independent experts)	Not specified; Authors from: Research Association Public Health Saxony and Saxony-Anhalt, Medizinische Fakultät Carl Gustav Carus, Technische Universität Dresden, Dresden, Germany	The basis for the systematic and comparative market analysis was defined by categories and respective subcategories/specifications outlined in Table 1...To examine the usability of currently available diabetes applications for the elderly, we performed an expert-based usability evaluation. With this method, usability experts put themselves in the role of potential or current users to examine products in terms of usability. We performed a summative evaluation as we exclusively included apps whose development was already finished.

6	Since the [search] keyword was generic, but the main target of our study was multifeatured apps dedicated to diabetes management for both patients and health-care professionals, we introduced some exclusion criteria. Apps were excluded from our analysis by the following criteria: they were a limited version (i.e., lite or free app) of an available fully featured version; they were supported only a single feature (e.g., insulin calculator only); they did not support any diabetes-specific data collection, archiving, and analysis for time-monitoring (e.g., glycated hemoglobin converter, pills reminder); their target was not diabetes self-management (e.g., generic health trackers, activity trackers, cooking apps, educational apps) or if diabetes self-management was an incidental-only element within the app; or if they were a content-consumption-only app (e.g., magazine, journal). Considering the fast evolution of the app market, we also excluded apps that did not receive any updates during the 12 months prior to the search...All apps meeting the inclusion criteria were used for our review. Among these apps selected for our study, a subset	According to our conceptual framework, we reviewed all the apps meeting our inclusion criteria by considering general features related to the mobile market (such as app pricing, app updates), diabetes-specific features including basic features (data logging, representation, and delivery), and advanced features (e.g., community services, insulin calculators) [26,34]. We considered whether the app was reviewed by one of the known reviewing initiatives.<br>Based on an existing tool for representing the benefits and weaknesses of medical apps, we created the pictorial identification schema/Diabetes Self-care tool, which specifically identified medical apps in the diabetes domain.	Not specified	a eHealthLAB, Dipartimento di Elettronica, Informazione e Bioingegneria, Politecnico di Milano, Milan, Italy<br>b Dipartimento di Ingegneria e Architettura, Università degli Studi di Trieste, Trieste, Italy<br>c Clinical Center for Neurostimulation, Neurotechnology, and Movement Disorders, Fondazione IRCCS Ca'Granda Ospedale Maggiore Policlinico, Milan, Italy<br>d Health Informatics Centre, Institutionen för lärande, informatik, Management och etik/LIME Department, Karolinska Institutet, Stockholm, Sweden<br>e Engineering in Health and Wellbeing Research Group, IEIIT-Istituto di Elettronica e di Ingegneria dell'Informazione e delle Telecomunicazioni, National Research Council (CNR), Milan, Italy	After reviewing the available apps, we identified the attributes relevant for diabetes self-care to be included in the new Pictorial Identification Schema.

	including the apps freely available from the Italian Apple app store were downloaded to test the apps directly. We decided to download only the free apps to let the users participate in the study without incurring additional costs.				

7	...apps were excluded if they: (a) had an estimated number of downloads below 100, (b) received user ratings below 4 (as done elsewhere [6, 7, 9]), (c) were not available on both iTunes and GP and (d) were free apps with limited functionality which is only unlocked by purchasing the full version (i.e., "freemium") [22]. In GP, freemium apps were filtered out as the database contains the variable "in-app purchases"; in iTunes in-app purchases were manually checked...the first author and a collaborator read the descriptions of the apps and applied further inclusion criteria. Apps were included if they addressed "weight management", which consists of both PA and dietary behavioural strategies [28], considering the limited role of PA and the predominant role of dietary strategies for effective weight loss [25, 27], and the importance of the combination of PA and diet for long-term effects on weight [28]. This allowed the exclusion of apps that focused only on PA and fitness. We also excluded apps that focused on other aspects of health (maternal health, mental health, etc.). Apps were excluded if their description was not available in English.	...two reviewers downloaded the selected apps and independently tested them using an iPhone 5S (iOS 9.0.2) and a Samsung Galaxy S4, GT-I9505 (Android 5.0.2). They first familiarised themselves with the app, used it for approximately 2 days, and independently documented features and evaluated the apps using online forms.	2	Not specified; Authors from: (1) Department of Health Promotion and Community Health, American University of Beirut, Riad El Solh, Beirut 1107 2020, Lebanon, and (2) Psychology Applied to Health group, University of Exeter Medical School, St Luke's Campus, Heavitree Road, Exeter EX1 2LU, United Kingdom	General and technical information were extracted for descriptive purposes.<br>Spearman's correlations were used to explore the relationships among popularity/ratings, total features, MARS scores, and number of techniques.<br>Independent samples t-tests and one-way ANOVAs (or nonparametric alternatives where indicated) were used to test differences among identified app categories.

	The selection process is summarised in Fig. 1.				

8	Each author reviewed the app descriptions she downloaded and one other author's apps, such that each of the 411 identified apps was reviewed twice. Initial review of descriptions occurred independently. Once all apps were reviewed, the two authors responsible for a particular app met to verify anddiscuss ratings. In the case of any discrepant ratings, the two authors together re-reviewed the app to come to a consensus rating. If a consensus could not be reached, the first author (JYB) made a final decision. Consensus between the two authors responsible for a particular app was reached for over 99 % of ratings and JYB made a final decision in five cases.	Review of downloaded apps for reliability was conducted by JYB and JY in a similar manner 5 months after the initial review of description pages. To reduce potential bias, the initial ratings of app descriptions were not referenced during thisprocess. Each author downloaded all of the randomly selected apps and independently reviewed\ each app. In order to ensure that all app features were explored, the authors clicked all possible links and entered data into all possible fields. Once all apps were reviewed, the authors met to verify and	2	JYB has an MS and JY has a BA. Both are from: Department of Psychology, Rutgers, The State University of New Jersey, 152 Frelinghuysen Road, Piscataway, NJ 08854, USA; and Institute for Health, Health Care Policy and Aging Research, Rutgers, The State University of New Jersey, 112 Paterson Street, New Brunswick, NJ 08901, USA	Applicability, Adherence to AADE7 guidelines and additional features were extracted and described. Descriptive statistics of all variables and chi-square analyses to assess associations between AADE7™ behaviors and developer type were run with IBM SPSS Statistics 19 (2010). Given the small sample size of apps developed by non-profit, education, and government institutions, a median split was used i chi-square analyses of differences in the number of AADE7™ behaviors promoted by different developer types. Tests of reliability between description-based and download-based ratings of the AADE7™ behaviors were conducted with Cohen's Kappa using the guidelines described by Cicchetti [15]: 0.75–1.00 = excellent; 0.60–0.74 = good; 0.40–0.59 = fair; and <0.40 =poor.

9	A total of 54 apps were included (from 1908 that were identified), among which there were 13 duplicates. Most of these apps were uploaded under the medical category. Seven had ratings <3.9 stars (out of 5). Only 4 apps had exceeded 1000 downloads, with a mean of 100–500 downloads. A total of 15 apps were aimed at health professionals, while 12 focused on patients. We identified 16 applications created from websites.	From the results, app descriptions and available screenshots as provided by site were analysed. We included all smartphone or tablet apps with HIV content, targeted at patients and the general population or aimed at healthcare professionals. We compiled lists of all identified apps to download. Any eligible app that was identified in only one store was searched for by name in the other store to confirm its exclusive availability. We downloaded and tested each eligible app. If an app had a free and a fee-based version, both versions were downloaded. Apple apps were download onto an iPhone 4 and an iPad 2 tablet, while Android apps were downloaded onto a Samsung Galaxy II phone. After download, an app was excluded if it did not feature pathophysiology content and pharmacotherapy information, was not available in English, or could not be used because of technical problems (after two attempts).	2	Not specified; Authors from: (1) Departament of Pharmacy, Valme Hospital, Seville, Spain and (2) Departament of Pharmacy, Complejo Hospitalario de Jaén, Jaén, Spain	[The authors developed their own criteria for all of their categories.] Results were tabulated using Excel 2007 (Microsoft, Redmond, Washington, USA). Descriptive statistics were used to summarise the results of the content assessment. We analysed averages and proportions. Inter-rater variability was measured with Cohen's kappa. All statistical analysis was performed using SPSS Statistics V.20.0 for Windows (SPSS Inc.).

10	Apps were excluded if the installation process was not successful or the app did not work properly…<br>All potentially relevant mobile apps by title and description were independently screened for eligibility by three experts (PPB, PK, ER) in the field of healthcare-related mobile apps. The subjective assesment of a mobile app's features by each reviewer introduces a source of bias to this study. In the attempt to mitigate this bias, we required that at least two reviewers agree with the inclusion of the app into the further analysis. Differences in judgement were resolved through a consensus process. The inter-rater agreement based on Cohen's Kappa statistic between the reviewers ranged between 0.77 (reviewer 1 vs. reviewer 3) and 0.90 (reviewer 2 vs. reviewer 3).	The evaluation of mobile apps features included five main categories (personal data, glucose and insulin therapy, nutrition, physical activity and additional features) with subcategories. A template containing the data that should be extracted was designed in the form of a Excel spreadsheet, which is further presented in Table 3. Each application was independently assessed by all three reviewers. The Cohen's Kappa coefficient ranged between 0.88 (reviewer 1 vs. reviewer 3) and 0.94 (reviewer 2 vs. reviewer 3), which indicates a high level of agreement.	3	Not specified; Authors from:<br>PPB: Faculty of Health Sciences, University of Maribor, Zitna ulica 15, 2000 Maribor, Slovenia<br>PPB, PK and ER: Faculty of Electrical Engineering and Computer Science, University of Maribor, Smetanova ulica 17, 2000 Maribor, Slovenia	Not explicitly described. Thematic analysis was used to identify similar capabilities and features of the apps.

11	Among the apps searched with keywords, those which were not relevant to smoking cessation were excluded. Apps were also excluded even if they had some relevance to smoking cessation in the following cases: (1) task management apps were dropped unless their primary purpose was to aid smoking cessation; (2) hypnosis apps for smoking cessation were disregarded because they attempt to exert a subconscious influence and are not appropriate to be analyzed within the frame of SDT; (3) apps developed for physicians to aid their medical treatment, rather than for general consumers, were also not included; (4) apps offering simulation of smoking were also not included unless they clearly stated their purpose as smoking cessation.	The apps searched and downloaded from December 2012 through January 2013 were analyzed The draft of the coding scheme was developed based on the aforementioned conceptualization of each variable, driven by SDT and the gain/loss framing theory. Based on this draft, the authors reviewed a part of the smoking cessation apps and revised the draft, taking into account their characteristics. This version of the draft was further fine-tuned through the process of coder training and establishing intercoder reliability.	2	Two undergraduate students fluent in both English and Korean served as coders. They became acquainted with the coding scheme through 2 training sessions.	Thematic analysis of SDT variables: descriptive characteristics, autonomy, competence, relatedness and goal contents and framing. Intercoder reliability and descriptive statistics were presented.

12	Reviewer: Not described<br>NK: could not find full text to confirm	Each app store was searched using keywords related to the commonest vascular diseases referred by general practitioners and seen by vascular surgeons at outpatient clinics.	Reviewer: Not described<br>NK: could not find full text to confirm	Authors from: Department of Clinical Surgery, Royal Infirmary of Edinburgh, Little France, Edinburgh, United Kingdom. 2Department of Clinical and Surgical Sciences (Surgery), School of Clinical Sciences and Community Health, University of Edinburgh, Edinburgh, United Kingdom	Data were grouped into subcategories by topic/target audience and summarized for clarity with results tabulated using Excel 2007.

13	The main inclusion criterion was that the application had a self-monitoring of blood glucose (SMBG) component. This inclusion criterion had the potential to preclude relevant applications, but in reality none of the excluded applications had a clear focus on diabetes. We settled on SMBG as the main inclusion criterion in order to filter out applications intended exclusively for medical professionals rather than patients, as well as other general health and lifestyle applications. We excluded applications without English-language user interfaces and those designed exclusively for health care professionals. We also excluded hardware-based solutions geared toward blood glucose tracking or insulin pumps only. Applications with their latest updates or publications prior to 2006 were excluded.	We analyzed the following features: (1) self-monitoring: (1.1) blood glucose, (1.2) weight, (1.3) physical activity, (1.4) diet, (1.5) insulin and medication, and (1.6) blood pressure), (2) education, (3) disease-related alerts and reminders, (4) integration of social media functions, (5) disease-related data export and communication, and (6) synchronization with personal health record (PHR) systems or patient portals. We installed the available applications and recorded the functionality in a spreadsheet (see Multimedia Appendix 1). For those that we were not able to install, we cross-referenced the function descriptions in published articles. We noted whether each of the functions required manual interaction with the user, or whether wired or wireless sensors were used to import data into the application automatically. We then compared the prevalence of features with the recommendations in several clinical guidelines (see Discussion section for references to guidelines). Guideline recommendations can provide a good basis for requirements analysis and specification during the design and development of diabetes applications.	1	TC: Norwegian Centre for Integrated Care and Telemedicine, University Hospital of North Norway, Tromsø, Norway, and Medical Informatics & Telemedicine Group, Department of Computer Science, University of Tromsø, Tromsø, Norway	Descriptive

14	The following data were extracted from the first 200 results found for each term in each app store (4 x 200): time and location of search, app name, developer name, ranking in the search results, cost, and classification. We considered 200 search results for each search term comprehensive as users rarely examine search results thoroughly [34].<br>Duplicate apps were removed from the 800 search results and the unique apps were classified as either alcohol reduction (apps that aim to reduce drinking-related behavior and those that track consumption), entertainment (drinking games, cocktail recipes, bar finders); BAC measurement; or other (apps not about alcohol, apps not in English, information for employers, etc).<br>Of the 91 alcohol reduction apps, we installed, examined, and coded all 51 free apps as users prefer apps that are free to download [35]. However, 10 paid apps were installed, examined, and coded as a sensitivity check of the BCTs included. The remaining paid apps (n=15), apps that could not be installed (n=5), or those that focused on hypnosis (n=10) were excluded (see Supplementary Figure 1).	For each app, BCTs were coded as 0: no evidence of BCT, 1: BCT present in all probability but evidence unclear; and 2: BCT present beyond all reasonable doubt and clear evidence	2	Both had MSc and were from the Department of Clinical, Educational and Health Psychology, University College London, London, United Kingdom	All statistical analyses were conducted using SPSS version 20.0. Frequencies, percentages, and associated 95% CIs were calculated for the categories of alcohol-related apps (alcohol reduction, entertainment, blood alcohol content, other), for each of the 41 BCTs, and for the mention of theory or the mention of evidence contained within the alcohol reduction apps.

15	Reviewer: blank NK: could not find full text to confirm	Summaries of elements of each app were coded line by line, which were then classified into categories based on common themes. This procedure was repeated as themes emerged until the data were placed into exclusive categories. This process resulted in two primary categories: those that “facilitated” alcohol use and those that aimed to “intervene.”	2	Not extracted	Not extracted

16	Apps were excluded if they were designed specifically for one medication type or a single disease. Lastly, those lacking a general description of functionality also were excluded.	App descriptions and available screenshots were analyzed for content and app functionality. To identify the apps that might have the most utility for patients that could be recommended by pharmacy practitioners, the authors developed a list of desirable attributes of these apps by consensus of all of the authors to evaluate them for comparison. Whether the app possessed each attribute was assessed based on each app's features described on their website or their respective product listing on their app source (e.g., iTunes). The relative desirability or usefulness of these features then were rated by the study authors using a three-point rating system (1, modest; 2, moderate; or 3, high) based on the perceived importance of each feature or characteristic. Apps were evaluated for each manufacturer claim that met the authors' scoring criteria, functionality of the reminder system, and ability to process reminders from the test medication regimen.	Not specified	Not specified; authors from: College of Pharmacy, University of Arkansas and the Translational Research Institute, University of Arkansas for Medical Sciences.	Rated apps based on weighted scale. 1, modest; 2, moderate; or 3, high) based on the perceived importance of each feature or characteristic

17	Based on the study protocol, each of the following criteria had to be met by the apps to be considered: functionality on both smartphone and tablet; 50 or more user reviews or ratings for all versions of the application on its respective store; average rating of higher than 50% based on user ratings for all versions of the application in its respective store; nutritional tracking capabilities; searchable food and nutrition database; macronutrient (e.g., carbohydrate, protein, fat) logging and/or tracking; inclusive database (i.e., contains data for basic, branded, homemade, and restaurant foods); option to use American units (e.g., pounds, cups, inches); available in English; and latest version of the app released January 1, 2014 or later. However, apps were excluded if they encountered a hard stop [designated by ‘‘NO (Stop)’’] on the evaluation form...The descriptions and other data provided for each of the individual apps on the respective app stores were used to ascertain the data used in determining which apps to include in the study.	Details such as version, ratings, and features for each of the resulting apps were documented in the study’s extraction form. These included apps were considered the final set and constituted the data for the review.	Not specified	Not specified; Authors from: (1)University of Tennessee College of Pharmacy, Memphis, Tennessee, (2) University of Mississippi School of Pharmacy, Oxford, Mississippi.	Descriptive

18	A diabetes app qualified as a self-management app if it performed one of the following functions: self-monitoring of blood glucose (SMBG), prandial insulin dose calculation or diabetes medication/insulin data tracking. Textbooks, cookbooks, strictly informational apps, and apps written in a language other than English were excluded from our analysis. Apps that did not function indefinitely (i.e. ceased to operate after a trial period) were also excluded.	Six different functions were examined for each app: SMBG, diabetes medication tracking, prandial insulin calculation, data graphing, data sharing and other data tracking (e.g. body weight, calorie intake). Each function was graded on a scale of 1–5, and the scores from the two researchers were then averaged.Judging focused on ease of use, user interface design, customizability, data entry and retrieval, integration of data into charts or graphs, and data sharing. The sum of the six function scores was an app's composite usability score (CUS) (scale 1–30). To account for well-performing apps which only contained a few functions, an average usability score (AUS) was also created.	2	Not specified; Authors from: Department of Internal Medicine, Mount Sinai School of Medicine, New York, USA; and Division of Endocrinology, Diabetes and Bone Diseases, Mount Sinai School of Medicine, New York, USA	Student's t-test was used in comparing the AUS and number of functions between free and purchased apps. The Mann-Whitney U test was used for all other comparisons between free and purchased apps. Correlations were assessed with Spearman's correlation coefficient (Graphpad Prism, La Jolla, CA, USA). Inter-rater reliability was calculated using the intraclass correlation coefficient (ICC), model 2 single form (StatTools.net, Hong Kong).

19	The authors included apps intended to support blood glucose (BG) monitoring and DM self-management in patients with type 1 DM (T1DM) and type 2 DM (T2DM).	The authors reviewed the current status of mobile medical apps with the goal of understanding the content, common design features, evidence for efficacy, and benefits as well as regulatory requirements governing mobile medical apps.	Not specified	Not specified; Authors from: Nebraska Medicine, Diabetes Center, Omaha, NE 68198, USA; Division of Endocrinology and Diabetes, Pediatrics, Stanford University, Stanford, CA 94305, USA; Sutter Health Integrated Diabetes Education Network, Quality and Clinical Effectiveness Team, Office of Patient Experience, Sutter Health, Sacramento, CA 95833, USA; Diabetes Research Institute, Mills-Peninsula Health Services, San Mateo, CA 94401, USA	Descriptive; Dicussion included presentation of results as answers to the following questions: (1) What Are the Common Characteristics of High-quality Mobile Medical Applications? (2) What Are the Benefits of Using Digital Health Applications (What Are the Efficacy Outcomes)? (3) What Are the Barriers to Mobile Medical Application Adoption? (4)What Are the Regulatory Requirements Governing Mobile Medical Application Use? (5) What Is the Spectrum of New Innovations Offered via mHealth? (6)What Is Desired for the Future of Mobile Medical Apps? (7) What Are the Directions for Future Research?
20	A total of 107 apps were identified using the search terms. Fourteen apps didn't have information about stroke, therefore excluded from the study.	The content of the applications was analyzed by two independent investigators (DD, AA). Each app was analyzed and classified on the basis of cost, target audience, type of information, validity, involvement of health-care agencies and usefulness based on audience reviews and ratings.	2	Not specified; DD: University of Texas Southwestern Medical Center, Dallas, Texas, USA.; AA: University of Nevada School of Medicine, Las Vegas, Nevada, USA.	Univariable analysis was performed using Pearson's chi-squared test or Fisher's exact test for categorical variables. A 2-sided Pvalue of <0.05 was considered significant. Kappa coefficient of agreement between two investigators was calculated.

21	...we reviewed the title, abstract, and full text (for peer-reviewed articles) using the following criteria.<br>To be included in the review, an application had to exhibit the following characteristics: Support for blood glucose monitoring as a minimum requirement; The patient as the intended primary user of the application, and; The application to be used as an enabler for diabetes self-management by supporting one or more of the self-management tasks. Applications excluded from the review exhibited any of the following characteristics: Duplicate applications; Applications where the sole purpose is to educate the patient about the disease; Applications without an English-language user interface, and; Applications intended exclusively for health care professionals.	For the purpose of evaluation, a set of 50 applications were randomly selected and independently reviewed by the authors with a joint probability of agreement of 0.98. We resolved any conflict by discoursing about the disputed applications and by revisiting the aforementioned criteria of inclusion and exclusion. We then independently reviewed another set of 50 randomly selected applications with a joint probability of agreement of 1.0. The remaining apps were reviewed by one of the authors; however, for each application review, another author did random crosschecking and validation.	1 (with random checking and methods to calculate joint probability)	Not specified; 1College of Business and Information Systems, Dakota State University, Madison, South Dakota<br>2College of Arts and Sciences, Dakota State University, Madison, South Dakota<br>3St. Elizabeth Healthcare Regional Diabetes Center, Covington, Kentucky<br>4Department of Internal Medicine-Division of Endocrinology, Sanford School of Medicine, Royal C. Johnson VA Medical Center, Sioux Falls, South Dakota	Descriptive

22	...two authors independently reviewing each of the English-language apps identified in the first step to select those primarily designed for people with eating disorders or for professionals helping those with eating disorders. Where there were inconsistencies, these were resolved by discussion.	Each of the apps identified in the second step was assessed. Their functions were grouped into four categories: (i) Provision of information; (ii) Self-assessment; (iii) Self-monitoring; and (iv) Provision of advice or treatment. Popularity of apps was also assessed using a platform called "Xyologic".	2	Not specified; both authors from the University of Oxford, Oxford, United Kingdom	Descriptiv e
23	Apps were excluded that were not in Portuguese and that were not about smoking cessation as judged by the reviewers.	Apps categorized based on function, fitting into the following categories: calculator, calendar, hypnosis, information tools, game, lung health monitoring, rationing, other.<br><br>Apps were judged according to adherence to "Treating tobacco use and dependence" guideline, made available in the publication.	Not specified	Not specified	Apps were scored for adherence to the guidelines. 21 criteria were assessed, where apps received a score of 2 if the criteria was fully addressed, 1 if partially addressed, 0 if not addressed.<br><br>Only descriptive statistics were presented.
24	Apps with 1 million installs or more were included in the analysis.	Apps were analyzed from both "nutritional" and "technological" perspectives. Nutritional: dietary intake, phenotype, physical activity, and Others. Technological: what emerging technologies have been included (e.g., barcode scanning)	Not specified	Not specified; authors from School of Systems Engineering, University of Reading, Reading, United Kingdom; and Hugh Sinclair Unit of Human Nutrition and Institute for Cardiovascular and Metabolic Research, Department of Food and Nutritional Sciences, University of Reading, Reading, United Kingdom;	Basic descriptive statistics regarding which apps had which features.

25	A first random sample selection of 50 apps (from a pool of 552 diabetes apps) in the app store was conducted by two reviewers independently with a Joint Probability of Agreement of 0.9 ...Disagreements on inclusion were resolved by revisiting the inclusion and exclusion criteria, or turning to a third reviewer. A second random sample selection of 50 additional apps was conducted with Joint-Probability of Agreement was 1.0. The primary author was responsible for reviewing the apps. Apps were downloaded and installed on reviewers' mobile phone to facilitate analysis. Two reviewers each checked the apps by operating every module, and extracting details including: name, version, operating system, cost and features.	We began with an identification of features and contents found in existing Chinese diabetes apps, proceeded to a comparison of evidence-based guidelines for self-managing diabetes in older adults with content in existing diabetes apps, and finally compared app features to usability guidelines and recommendations for older adult populations.	2	Not specified. Gao, Zhou, and Liu – School of Nursing, Second Military Medical University, Shanghai, China; Wang and Bowers – School of Nursing, University of Wisconsin-Madison, US	We employed the usability assessment criteria developed by Arnhold et al [20], that consists of four main criteria (comprehensibility, presentation, usability and general characteristics), 11 subcriteria and 18 items with 5 Likert-scales and dichotomous scales....Each item was independently scaled and average scores were obtained. The inter-rater reliability was measured using intra-class correlation.<br><br>Descriptive statistics were used to present the apps.

26	Applications were included if they were written in English, medication related, and last updated in 2014. Applications were excluded if they lacked a ‘‘Description’’ under the ‘‘Details’’ section of the App Store, were specific to a single medication (e.g., birth control) or single disease (e.g., HIV, COPD), had health-related functionality other than medication adherence such as blood pressure or blood sugar monitoring, were tailored to countries outside of the United States, specific to members only (e.g., insurance plans), or utilized for veterinary medicine, and if they lacked at least one ideal application feature.	The Apple iTunes App Store highlights features of an application using a description and screenshots for the user to identify if they are interested in downloading the application to their mobile phone. We used each application’s description and screenshots to identify available application features.	Not specified	Not specified. Department of Clinical Pharmacy, College of Pharmacy, University of Michigan	Descriptive

27	Apps were selected based on 29 topics identified from the following: (1) patient education topics recommended in the Canadian Diabetes Association 2013 Clinical Practice Guidelines for the Prevention and Management of Diabetes and (2) the seven self-management behaviors identified by the American Association of Diabetes Educators. The topics were grouped into five categories: (1) healthy eating, (2) physical activity, (3) self-monitoring, (4) problem solving, and (5) healthy coping.	In March 2014, a series of search strategies was used to identify top-rated iPhone and Android health apps, representing 29 topics from five categories of type 2 diabetes self-management strategies. The senior author (KH) assessed the most popular apps found that addressed these topics using the Behavioral Theory Content Survey (BTS), which is based on traditional behavioral theory. A tool to assist decision making when using apps was developed and trialed with health professionals for ease of use and understanding.	1	The primary author Kelli Hale lists a BAppSc and BHlthSc(Nutr&Diet)(Hons), and is from the Centre of Dietetics Research, School of Human Movement and Nutrition Sciences, University of Queensland, St Lucia, Australia	The Behavior Theory Content Survey assesses interventions for the use of 20 strategies shared by four key behavioral models/theories: (1) Health Belief Model, (2) Theory of Planned Behavior, (3) Transtheoretical Model, and (4) Social Cognitive Theory. The strategies are listed individually as they are common to more than one theory. Each intervention strategy is scored out of 5 as it is rated dichotomously for the inclusion of the following five dimensions of user interaction: (1) provides general information or guidelines, (2) assesses current practices or use of strategies, (3) provides feedback on assessment, (4) offers general assistance on behavior change, and (5) offers individually tailored assistance in response to assessment and feedback. The levels are hierarchical as level 5 (individual advice) is thought to be more effective than level 1 (providing general information). The BTS is the sum of scores for all 20 intervention strategies; the maximum BTS score is 100, representing 20 strategies, each of which are scored out of 5 to indicate the level of interactivity.

28	Once all apps were identified using the previously described protocol, the first author reviewed all apps identified via keyword searches and excluded those apps that met the following exclusion criteria: 1) did not target diet tracking, 2) did not include taking or posting pictures of food for the purpose of self-monitoring diet, and 3) not in English. The first author compiled a list (on October 6, 2015) including all of the relevant apps meeting inclusion criteria from each of the search terms used.	The reviewers gathered general information about the apps including: the current version, behavior targeted (eg, diet), available platforms, user satisfaction score (average number of stars received on a 5-point scale with 1 as the worst and 5 as the best), ability to connect to social media (assessed as ability to link posts with a social networking site such as Facebook), and the number of downloads (available for Android apps only) using the App Store description page. Both the App Store description pages and the apps themselves were used to determine how feedback on foods posted was provided to users including: crowdsourcing (eg, allow users to rate other users' food photos for healthiness) or collective feedback from other users (eg, in the form of a comment or message), professional feedback (provided via a health coach or registered dietitian nutritionist [RDN]), or no feedback provided.	2	Sarah Hales, PhD, MSW, LMSW,1 Caroline Dunn, MS, RD,1 Sara Wilcox, PhD,2 and Gabrielle M. Turner-McGrievy, PhD, MS, RD1 1Department of Health Promotion, Education, and Behavior, University of South Carolina, Columbia, SC, USA 2Department of Exercise Science, USC Prevention Research Center, University of South Carolina, Columbia, SC, USA<br>Sarah Hales, PhD, MSW, LMSW, Department of Health Promotion, Education, and Behavior, University of South Carolina, Columbia, Discovery I Building, Room 529, Columbia, SC 29208, USA.	The reviewers coded each app to determine the number of self-regulation techniques and other behavior change techniques (selected from Michie et al)25 that were included in the apps reviewed (coded as 1 for the presence, and 0 for the absence of each technique).

29	Apps not pertaining to the support of smoking cessation were removed. A final de-duplicated list was generated to identify the total number of unique and relevant apps offered for smoking cessation. The top 50 relevant recommended apps per search term were reviewed for the previously identified scientifically supported apps identified during phase 1, and results were documented. Only the top 50 apps were chosen for review for the sake of efficiency and to best represent real search behavior (i.e., minimize scrolling, focus on the first page of results).	The Preferred Reporting Items for Systematic Reviews and Meta-Analyses (PRISMA) guidelines were used to review apps.	Not specified	Not specified. Department of Emergency Medicine, University of Massachusetts and Department of Emergency Medicine, Psychiatry, and Quantitative Health Sciences	Descriptive

30	English-language apps with the stated capability to generate medication reminders were included in the analysis.	Two investigators independently analyzed the developers' descriptions and available screenshots in the online marketplaces to determine if an app possessed any of the 28 author-identified features. Investigators cross-verified initial app scores available on multiple platforms. The sum of the weighted scores was used to calculate an initial score (maximum of 68 points). Apps were ranked by means of these initial scores based on the developers' descriptions.<br><br>The top 100 highest-ranking adherence apps were then subjected to user testing to compare their quality against the developers' descriptions by installing the apps on smartphones. Adherence apps that could not be installed by at least one investigator or failed to generate reminders were excluded from further user testing. Two investigators tested each app independently over a 7-day period.	2	Scholars, clinical practitioners, health literacy specialists, and student pharmacists	Descriptive analytics and scores: Each feature was evaluated on a nominal scale and assigned a weighted score based on the importance within the respective domain (1, modest; 2, moderate; and 3, high).

31	Of the downloaded apps (n = 273), n = 48 were excluded from analysis, because they either were no longer available online by the time the second rater completed the app coding (73%), did not function properly (19%), were not in English (4%) or were advertisements for other apps (4%).	Each app's number of downloads and user rating (if available) were recorded from Google Play as measures of app popularity, in line with previous research, 12 and user-perceived quality, respectively. User ratings were provided on a 5-point scale, with 1 being the lowest rating and 5 the highest.	2	Not specified; authors from: Center for Addiction Medicine, Harvard Medical School and Department of Psychology, Suffolk University, Boston, MA and School of Education, Johns Hopkins University, Baltimore, MD	We calculated means with standard deviations and total n with percentages for the coded variables. In order to identify qualities of the apps which were associated with the popularity and user-rated quality of the apps, we used univariate regression models. Given the descriptive nature of this content analysis, we used an explorative approach, and thus did not correct P values for multiple testing. We used logistic regression to test predictors of popularity (ie, >10 000 downloads vs. fewer, the top 20% of the rated apps), and linear regression to test predictors of user-rated quality (ie, average number of stars per app). Analyses concerning the quality of the apps were restricted to the apps that had star ratings (77% of the apps).

32	Each search only shows 200 app results. Due to restraints in time and resources, the number of apps included had to be restricted. The first ten apps passing the pre-screening from each search term were included, giving 40 apps in total. Following identification, the apps were downloaded and evaluated again based on the same inclusion 96 and exclusion criteria as stated above. At this point some of the apps were excluded, and therefore a second stage of searches and screening was performed to meet the study's aim of evaluating 40 apps, ten from each search term. This second search was performed on 9 June 2015. Five apps were independently evaluated by another assessor in order to determine the repeatability and relative validity of the assessments.	Each app that met the inclusion and exclusion criteria was used by the author (CH) to identify the functions and BCTs included. The results were recorded in a data extraction form (Table 4) recording the functions and BCTs included in each app.	1	Not specified; Charlotte D Hoppe: School of Food Science and Nutrition, University of Leeds, Department of Nutrition and Dietetics and King's College Londo	The results were analysed using the statistical software Stata/IC (Release 13.1; Stata Corp, College Station, TX). T-tests were performed to assess the difference in mean number of functions, number of BCTs, overall score, price and user rating according to inclusion of 'optimum BCT', price (free or paid) and user rating. For the latter, user rating, normally ranging from one to five, was divided into the following two groups; low=1.0-4.0 and high=4.1-5.0. The uneven division of user rating was due to average app rating for the majority of apps being greater than 4. Regression was performed to see if there was a relationship between number of functions, number of BCTs and overall score versus Diabetes apps with behaviour change techniques Page 6 of 14 price (£) and user rating. Regression models for price adjusted for user rating and vice versa. Cohen's kappa was calculated to determine the inter-rater reliability from the duplicate extracted data.

33	Of the 175 mHealth applications, 165 were excluded for the following reasons: 61 applications were not in the English language (e.g. applications in Chinese, Arabic, Spanish and French), 34 applications were irrelevant to asthma (e.g. an application on coronary heart disease), 22 applications were physician focused and 41 were paid applications. In all, 22 applications made it to the next stage of the review. After careful evaluation, another seven mHealth applications were excluded because they were not closely related to asthma.	Each Application was coded into one of the following categories: (1) Basic facts about the nature of the condition; (2) The nature of treatment: relievers and preventers; (3) Allergen and trigger avoidance; (4) How to use treatment; (5) Self-monitoring and assessment skills; (6) The role of a written, personalized action plan; (7) Recognizing and responding appropriately to acute exacerbations; or (8) Personalizing the definition of good asthma control. Each application was coded by the researcher and cross-checked with another researcher. There were no disagreements between the coders.6,7,14 Asthma applications were also coded for their level of adherence to the Health On the Net (HON) Foundation principles for health information on the Internet as adapted by Huckvale et al.	2	Not specified; authors from: King Saud Bin Abdulaziz University for Health Sciences, Kingdom of Saudi Arabia; Ministry of National Guard Health Affairs, Kingdom of Saudi Arabia; Monash University, Australia; King Saud University, Kingdom of Saudi Arabia; King Saud Bin Abdulaziz University for Health Sciences, Kingdom of Saudi Arabia; Ministry of National Guard Health Affairs, Kingdom of Saudi Arabia; King Faisal Specialist Hospital & Research Centre, Kingdom of Saudi Arabia	Descriptive

34	Apps were screened by two authors (KH and MC). Apps were excluded if: not available through an approved device marketplace; explicitly disclaimed use for a health-related purpose; could not be downloaded because of country restrictions that orevented access in the UK; could not be used because of technical problems, after two attempts	Assessment was performed by two authors. Basic details were extracted into a standard form reviewer recorded their responses in a structured form. These were compared and any discrepancies were resolved by discussion.	2	Not specified; authors from: Global eHealth Unit, Department of Primary Care and Public Health, Imperial College London, St Dunstan's Road, London W6 8RP, UK., and Engineering Design Centre, University of Cambridge, Trumpington Street,<br>Cambridge CB2 1PZ, UK.	Descriptive

35	Obviously irrelevant apps were eliminated by reviewing app store descriptions and screenshots to identify apps that were either unrelated to diabetes self-management or for which no calculator could be present, for example, diabetes eBooks.	Apps were assessed using a standardized method to examine each component of the calculation process. Expected inputs and outputs, supported unit systems, terminology, and any supplementary app features were characterized by inspection. Simulated data were used to define behavior in response to missing and extreme input values. Where the formula used for calculation was not displayed in the app or in associated documentation, the developer was contacted. For those apps where a formula was ultimately identified, performance was assessed using a set of test cases generated by permutation of the range of possible values for each input parameter. Where present, clinical disclaimer text was extracted and coded to identify statements advocating discussion with a healthcare professional prior to calculator use and the role of personal judgment in interpreting generated results.	2	All apps were assessed by a clinician-researcher (KH) and a second reviewer (either JTP, a mobile health researcher, or SA, a public health researcher).	Fisher’s exact test was used to calculate the two-tailed probability of an association between issue prevalence and platform or distribution model. A significance level of 0.05 was prespecified. Statistics were computed with R (Version 3.0.0) using the package exact2x2 (Version 1.4.0).

36	Since our primary focus of attention was CBT or BA only those apps that offered this type of treatment were downloaded for full evaluation. When both a paid and free version of an app was available, the version requiring payment was purchased and used, while the free version was excluded. This was done to ensure that the most comprehensive version of the app was considered.	The apps retrieved by our searches were categorized by two independent reviewers (AH, JC) according to the type(s) of support that they offered to the users. The categories, defined a priori, included: self- tracking tools, education, social support, CBT/BA treatment, state induction, diagnostic/screening tools, and miscellaneous.	2	To evaluate whether the app actually offers CBT or BA, an experienced academic CBT clinician (SR) evaluated the apps for their level of fidelity to theoretical CBT and BA principles by exploring what extent the apps included the core ingredients of these models. The evaluator has extensive experience in training CBT therapists and devising CBT clinical programmes. The two reviewers are from: Center for Research in Family Health, IWK Health Centre, Halifax, Nova Scotia, Canada	For usefulness: The expert evaluated each app against each core ingredient on a 0–2 scale where 0 meant that the core ingredient was not integrated at all into the app, and 2 meant that the core ingredient was completely integrated.<br>For usability: The usability expert rated each app on a scale of 1 to 5 (1 = poor, 5 = excellent) against each usability heuristic.<br>Final anaylsis: Basic summary statistics including counts and percentages were used to describe the characteristics of the apps. Spearman's correlation coefficient was used to explore whether a relationship may exist between the adherence of the user interface to Nilsen's principles of usability and adherence to the core principles underlying CBT and BA.

37	We excluded the mobile apps and systems that could only be considered as educational or informational tools, meaning those that did not provide any direct functionality for the self-management of diabetes-related issues.	Categories, upon which we extracted relevant app information, were based on previous research a literature reviews and further elaborated upon via iterative brainstorming among the coauthors. The agreed upon categories are as follows: Diabetes-related features (scored on a scale of 0-2); Popularity and presence in social media; Availability; Interoperability and 'shareability', User friendliness; Quality assurance and regulatory oversight; and Research based.	Not specified.	The coauthors have extensive research experience with mobile applications and have a multidisciplinary background ranging from healthcare and business, to health informatics, statistics, computer science and electrical engineering.	Descriptive
38	To determine eligibility for full review, two Master's level coders catalogued basic information for each app link retrieved. This approach to app screening has been used in other reviews . To be eligible for full review, apps had to be in English and include text related to cessation either in the Facebook App Center overview or within the app.	Eligible apps were installed using the native Facebook platform on a personal computer or the iPhone platform for mobile-only apps. Coders used apps over 3 days with at least 3 logins to ensure all features were utilized and coded. Apps were coded for publisher/developer type, cost, and content features (interactive, informational, and social). Operational definitions of content features were developed prior to coding and were noted as present or absent.	2	Two [public health] Master's level coders catalogued basic information for each app link retrieved.	Frequencies and descriptive statistics were used to characterize apps. For each adherence index item, the proportion of apps receiving a 2 (fully present) by at least one coder was calculated (average score≥1.5; as in). The relationship between publisher/developer source and app type on the adherence index summary score was examined. All analyses were performed in SPSS Version 21.

39	Reviewed the description of each application.	4 nurses each evaluated 30 apps, 148 were catrogrized into purpose, 104 for providing health information on obesity management' were further analyzed.	1 per app	Nurse informatists who majored in nursing informatics in graduate school and had experience of developing healthcare mobile applications were trained until an inter-rater reliability of 1.0 was reached	Descriptive statistics; A t-test was used to compare the quality scores relative to price<br>Pearson correlation coefficient was used to study the correlation between user rating and health-information quality
40	Apps were included if they directly focused on treatment, psychoeducation, assessment, or provided general information about eating disorders or disordered eating behavior. Apps were excluded if they did not specifically address disordered eating or focused solely on tracking food intake or weight loss.	Coding and thematic analysis yielded three main categories of apps for eating disorders: 'Treatment,' 'Psychoeducation,' and 'Other.' Only treatment-focused apps were considered for the purposes of this review.	2	Not specified; authors from: Drexel University Department of Psychology, Philadelphia, USA	Descriptive

41	Since users are most likely to pursue the top search results, only the top 50 apps for each search term were included in the analysis. Apps repeated in the results of the two search terms were included only once. A total of 200 apps were screened in this manner and 107 unique apps were included in the analysis. All applications in English that contained information or tools to manage systemic HTN were eligible for analysis.	We recorded data on total number of reviews for all versions of the app using publically available data on Apple iTunes and Google Play. The total number of downloads are reported only by Google Play in the form of an ordinal variable with nine different categories ranging from 50–100 to 1 million–5 million; this download information was available for a total of 50 apps included in the study. We recorded major functional characteristics of each app in the following non–mutually exclusive domains including hypertension education, tracking function, tools to promote medication adherence, whether the app can transform the smartphone into a medical device, and whether access to support groups and patient forums was facilitated.	Not specified	Not specified; authors from: Department of Medicine, Cambridge Health Alliance, Harvard Medical School, Cambridge, MA, USA; Department of Medicine, All-India Institute of Medical Sciences, New Delhi, India; Department of Medicine, University of Texas Southwestern Med Center, Dallas, TX, USA; and Division of Nephrology, Department of Medicine, Beth Israel Deaconess Medical Center, Harvard Medical School, Boston, MA, USA	To test the functional characteristics of an app that are associated with a higher number of downloads, we built an ordinal logistic regression model. In order to understand the app characteristics that are associated with the most favorable ratings by users, we fit a model for count data.

42	The sub-setof those apps which involved some elements of interaction with the user and comprised of one or more self-help strategies for dealing with depression were downloaded for further evaluation in line with the specific objectives mentioned earlier.	278 apps were identified in the first step and these spanned a wide range of categories. Information on coping with depression and stand-alone screening tools formed the two largest types of free apps. Features of interactive self-care apps were reviewed further and this exercise showed that less than 10% of the apps incorporated explicit delineation of their scope of initial screening for suitability.	2	Not specified; authors from: Department of Clinical Psychology, Bangalore, India	Apps were examined for presence of the following features: a) explicitly defined scope (there are further details about this in the article), b) whether the app provided direct encouragement to seek professional help when needed was coded (present/absent), c) the same strategy was used for coding presence/absence of guidelines for dealing with crisis (beyond a mention in the terms and conditions), d) an attempt was made to document whether the app contained information for users regarding its theoretical and/or research basis because this can be of help to users for making informed choices, e) in addition, presence of a privacy statement and terms of use was documented too as these help users understand the potential risks involved in using the app, including limits to confidentiality, etc.<br><br>The third step entailed classification of therapeutic content. The nature of the sections in the apps and the various exercises incorporated were scrutinized for this purpose.

43	Following download, each app was opened and assessed independently by the two reviewers to confirm eligibility. In the current review, free and paid-for apps containing content related to suicide were included if they could be downloaded via the official Android and iOS stores. Apps were excluded if they: contained no “active” or “interactive” suicide prevention content; referred to suicide non-literally, for example in branding, or music titles; referred specifically to self-harm with non-suicidal intent; were related exclusively to depression, bipolar disorder, or other mental health conditions, unless suicidality was explicitly mentioned; or were not in English, or included character sets which did not display correctly.	The content and features of the apps were then independently reviewed for both harmful and suicide prevention content. The reviewers used a custom coding scheme (see Data Items section), and coded the interventional components directly into a database created for the review.	2	Not specified; authors from: Black Dog Institute, University of New South Wales, Sydney, New South Wales, Australia, School of Psychiatry, Faculty of Medicine, University of New South Wales, Sydney, New South Wales, Australia	Descriptive; the reviewers used a custom coding scheme

44	The eligibility requisites used for this review were the following: apps not in English or in the language of the place where the search was executed (Spanish) were dismissed, the same as those with their summary in a different language from the two mentioned. Only applications focused on cardiac issues were studied. Games, music, apps for cholesterol management or losing weight, apps for animals, and apps for congresses or conferences were dismissed, but apps related to hypertension were included in the study.	Once the selection of cardiology-related apps was complete, the authors convened again to sort the apps by their purpose in different types, by reading the summary and explanation given by the stores and downloading them when the explanation specified was not clear enough. In these cases, the smartphones used were an iPhone 4 if the app was designed for iOS and a Samsung Galaxy S SCL GT-I9003 in the case of an app for Android. Finally, revisions were done in order to shorten the classification, similar to the process followed in the literature review.	Not specified	Not specified; Authors from: University of Valladolid, Department of Signal Theory and Communications, and Telematics Engineering	Descriptive; Mean, median and mode of prices of commercial apps by store

45	Two applications for each condition, one for each review, were selected for an in-depth analysis. For the in-depth analysis, two applications were chosen for each condition: one obtained from the literature research and the other from the commercial apps review. If the app studied was available in stores, it was downloaded and personally tested on an iPhone 4 in the case of an iOS app or a Samsung Galaxy S SCL GT-I9003 in case of an Android app. For apps available on the market, one of the authors downloaded them for a joint evaluation at the meeting.	To evaluate the papers, after reading them individually, the authors convened to discuss opinions and fill in a table of features. For apps available on the market, one of the authors downloaded them for a joint evaluation at the meeting.<br>For the analysis of the commercial apps, the procedure followed was similar to the one developed with the research papers. The authors downloaded them on the mentioned mobile phone Samsung Galaxy S before meeting to study the apps together and complete the previously initiated table of features.	Not specified	Not specified; Authors from: University of Valladolid, Department of Signal Theory and Communications, and Telematics Engineering	Descriptive

46	A set of 50 apps was generated for each keyword term. The 2 sets of apps were merged. Because 17 duplicates existed, a list of 83 apps was generated. A prefiltering of the apps was performed by the study team to discard apps that were clearly not exercise prescriptive. The agreement among the study team was unanimous for discarding 22 apps from inclusion into the review. Of the 61 apps that were reviewed, 31 additional apps were discarded because exercise prescriptive programs were not provided, and therefore, did not meet the study inclusion criteria.	The final set of 30 apps was scored for quality of content against the guidelines of exercise prescription of the ACSM.	3	Not specified; authors from: Department of Health Outcomes and Policy and Department of Orthopaedics and Rehabilitation	An overall quality score and subscores for the 3 components of aerobic exercise, strength/resistance training, and flexibility were generated. Basic statistics were computed (arithmetic mean, SD, maximum for the set of the final 30 apps). A threshold quality score was established to indicate whether each app provided at least half of the content of the ACSM guidelines for the overall app score and the component subscores (3/6 points for aerobic exercise or strength/resistance components, and 1/2 points for flexibility). Inter-rater reliability was assessed using the Krippendorff alpha coefficient [24]. The results were visualized using box-plot mapping. Statistical analysis was performed using R software (version 3.1.2).
47	Based on these multiple searches and EIN physician recommendations, our search resulted in approximately 1200 apps. After excluding games, and consumer and non-English apps, these authors previewed the apps and applied exclusion criteria.	A summary of 24 selected apps was grouped into categories: Pediatrics, Infection Prevention/Control, Antimicrobial References, and Clinical References; 12 apps were not previously reviewed.	Not specified	Not specified; authors from:Division of Infectious Diseases, Department of Pediatrics	Descriptive

48	1,041 apps found through systemic searching and of these, 612 were unique. Based on exclusion criteria, 446 were excluded.	The content and functionality of each app was examined on a mobile phone or tablet through extensive exploration of the menu options. The downloaded apps were screened and coded for BCTs using the BCTTv1.	2	Not specified; authors from: School of Psychology, National University of Ireland, Galway, Ireland.	BCTs were coded with a "0" if absent and "1" if present. Inter-rater reliability was calculated. Statistical analysis was conducted in March 2015 using SPSS, v2.
49	Apps included for review were those that provided a bladder diary as part of their function. Apps that did not include a bladder diary and were merely educational, provided advice, alternative treatments, those relating to gynaecological or oncological content, product advertisements, entertainment, adult related material, games, and non-English apps were excluded from this review	All apps were downloaded and installed to the appropriate mobile phone. Apps were rated by the reviewers based on pre-set rating criteria. A final list of apps was compiled with details of mobile platform, number of reviews/ratings, pre-purchase rating, date of last update and cost of app.	4	Apps were rated by a paediatric urology consultant, fellow, registrar, and resident medical officer	Descriptive

50	Apps were included if: (1) app included a medication reminder feature; (2) app was available in the English language; and (3) app was designed for use with human medications.	Phase 1 was app selection. During phase 2, two raters independently downloaded and evaluated the presence or absence of 15 content features for each app by using each app over the course of a day. In addition, app-written descriptions were reviewed for any mention of the app being designed for use by children, adolescents, or families. Content features of focus were those known to be useful in pediatric adherence-promotion trials including medication reminder features (five features; e.g., able to set time-based medication reminder), behavioral modification features (five features; e.g., app provides reinforcement for logging medication as taken), and educational features (five features; e.g., app provides information about the benefits of adherence. Phase 3 of coding involved examining the usability of a subset of apps that had the highest proportion of content features (defined as apps scoring >1 SD above the mean on number of content features). App usability was evaluated using the engagement, functionality, and aesthetics subscales of the MARS.	2	The two reviewers were trained. Training involved having raters code sample apps using the procedure identified previously. Raters were required to evidence 90% agreement or higher before beginning Phase 2 coding.	Percentage of apps containing particular features related to each study objective (educational messages, behavior change features, design for children). Descriptive statistics on MARS scores.

51	The inclusion criteria for the apps to be included in the review were that the app had to be in English, primarily related to the disease gout, designed for patient use and incorporate elements of disease monitoring and/or patient education. The App Store description of each app was then read and assessed against these inclusion criteria.	Two investigators (AN, DK) independently assessed the six apps that met the inclusion criteria, separately, against each of the recommendations from Table 2. For a recommendation to be considered present, the app needed to include a function or educational material (e.g. an informational statement or section) for that specific recommendation. Once again, the investigators met to compare results and came to a consensus after discussion.	2	Not specified; authors from: Department of Clinical Pharmacology and Toxicology, St. Vincent's Clinical School, Centre for Health Systems and Safety Research, School of Medicine, School of Public health	Thematic analysis
52	Apps were included in the review if the following criteria were met: (1) developed for BD, (2) aimed at consumers and/or caregivers, (3) available for download through official Android/iOS app stores, and (4) available in English.	Descriptive characteristics related to the following features were extracted: (1) app accessibility, including platform, price, need for network connectivity, number of downloads, and average user-scored star rating for the app's entire history, (2) primary function, such as app store classification, app function (see below), and target audience, (3) app source, including provider type and details, crisis information, disclaimer presence, and app update schedule, and (4) privacy and security, presence of an accessible privacy policy, and the ability to password protect data.	2	Not specified; Authors specialize in Psychiatry. Clinical Psychologist reviewed findings.	Descriptive statistics were used to summarize the results of the app assessment. Pearson product-moment correlation was performed to examine relationships between comprehensiveness and quality of information, and the average user rating of an app. Mann Whitney U tests were performed to examine differences in comprehensiveness and quality of information by app price. Findings were compared thematically against evidence based guidelines.

53	The five free, and five paid, most downloaded apps from 'Lifestyle/Health' and 'Fitness' categories of the five app stores for the same countries (total 500 apps) were identified for more complete description, using App-Annie software.	Basic descriptions of the apps were provided using the specific content of focus (e.g., fitness, diet, etc.), and the total number of apps available in each market were stated. Apps were searched to determine whether any input had been provided by government agencies or whether formally endorsed guidelines were used.	Not specified	Not specified; authors from:Biogov, Universite Catholique de Louvain, Collège Thomas More, Louvain, Belgium; and Human Nutirition, School of Medicine University of Glasgow, Scotland.	Descriptive
54	Apps in the top 100 paid and top 100 free "health and fitness" categories of iTunes and the Android Market were searched. Inclusion in the top 100 is determined by the number of times the app has been downloaded. Only weight-loss mobile apps that included weight, diet, and physical activity self-monitoring were included in the present study.	Apps were downloaded and compared to 20 strategies deployed in a previously completed large scale lifestyle intervention.	2	Not specified; authors from: the University of Massachusetts Medical School (Pagoto, Jojic), Worcester, Boston University, (DeBiasse), Boston University School of Medicine (Mann), Boston, Massachusetts; and the Rosalind Franklin University (Schneider), North Chicago, Illinois	For each behavioral strategy, the percentage of apps that included it was computed, in order to demonstrate the extent to which each strategy was employed across weight-loss apps. Then, for each app, the percentage of the total number of strategies included was computed. Using ANOVA, paid apps were compared to free apps, on the measure of percentage of total strategies included.

55	We screened potential weight loss and smoking cessation apps to identify a final list of 120 Android and Apple apps (four groups of 30 apps each) based on their focus on the topic, price (all under $4), being in English language and download popularity as estimated by a relevant website (xyo.net).	Each app was examined by two assessors and was rated against a published "Mobile App Rating Scale" (MARS),19 (45% of the total score); in terms of weight loss/smoking cessation as appropriate (45% of the total score); and cultural appropriateness criteria (10% of the total score). We designed these other criteria (in addition to the MARS) based on relevant New Zealand literature.	2	Not specified; corresponding author (Nick Wilson) from: Public Health, University of Otago, Wellington	Basic report of scoring on MARS, and self-developed scales (weight loss, smoking cessation, cultural appropriateness).

56	We assessed all apps found by using the keywords ‘alcohol addiction’ (62 apps), ‘alcohol help’ (27 apps) and ‘stop drinking’ (21 apps). For the keyword ‘alcohol’, the first 40 apps were analyzed to provide a more comprehensive list of apps. This number was chosen on the basis of previous studies on Internet users showing that people rarely search beyond the first 20 retrieved results. Apps not related to problematic alcohol consumption (reducing alcohol consumption) as a main topic were excluded. Further exclusion criteria were as follows: the app could not be found at the moment of analysis, the app could not be downloaded after 3 attempts, the app was a book or an article, the app was not in English or the app consisted only of a contact address.	We evaluated the apps by using tools from previous studies and modified instruments from quality evaluation studies of websites , as well as instruments represented in various studies on the quality of smartphone apps.	3	The study involved 3 trained evaluators (L.P., M.V.S. and Y.K.). Raters completed an in-group practice run of a set number of materials, which were checked (with a standardized coding process for each component) and discussed before the evaluators participated separately in a formal assessment process.	After an initial exploratory analysis involving the calculation of proportions, as well as means and SDs of the above-mentioned measures, we compared paid apps with free apps in bivariate analyses using parametric tests (t test, chi-square or Fisher’s exact test) or non-parametric tests (median test) when appropriate. Next, we computed prediction models by using multiple linear regressions for 2 outcome variables of interest: content quality and self-help.

57	The first 20 apps appearing in each search and their accompanying descriptions were recorded into a database. We chose 20 as most users typically view one page of search results.	App characteristics were listed based on (a) general app characteristics (such as number of downloads), (b) relevance to mental health (diagnoses or symptoms, etc.), (c) "apparent purposes", (d) approaches for symptom relief, (e) information supporting app use or diagnosis, and (f) sescriptive terms used for depicting qualities of the app in app descriptions.	2	Not specified; Authors from: Children's Hospital of Pittsburgh of UPMC, University of Pittsburgh School of Medicine, Pittsburgh, Pennsylvania; Division of Adolescent and Young Adult Medicine, Department of Pediatrics, University of Pittsburgh School of Medicine, Pittsburgh, Pennsylvania; School of Social Work, University of Southern California, Los Angeles, California; University of California Los Angeles, Los Angeles, California; and RAND Corporation, Pittsburgh, Pennsylvania.	Content analyses were conducted.

58	We searched the categories medical as well as health & fitness to maximize our capture of apps that may be relevant to dementia by focusing on topics such as cognitive health, which are often classified as “health & fitness” rather than medical apps. Second, we determined which apps collected user-generated content, with the intention of excluding purely informational apps. If any of the following words were included in the App Store description we assumed that the app collected some form of user data: analyze, assess, collaborate, communicate, data, email, game, graph, location (GPS), journal, keep, measure, monitor, notes, photos, play, post, predict, progress, question, questionnaire, rate, record, report, research, results, save, score, screen, send, share, statistics, store, survey, take, test, tips, tool, track, train, write.	Regarding general characteristics, we examined whether privacy policies covered the app in question (rather than the developer or Web site in general), explicitly mentioned safeguards for protecting user data, and distinguished between how individual-level and aggregate data would be handled. To assess for the existence of safeguards, we interpreted any mention of “encrypt, encryption, physical security measures” or “an established records retention and disposal system” as an indication that a mechanism for protecting user-generated content was in place.<br>Regarding the protection of individual-level data, we documented whether privacy policies disclosed the collection of Internet protocol (IP) addresses or unique device identifiers (UDIDs), or whether they were explicit in their ability to store cookies on user devices. We noted whether policies admitted to sharing information with business partners or third parties, as indicated by references to the following terms: “advisers, affiliates, any other party, business partners, contractors, partners, partner companies, service providers, third parties.” Similarly, we documented whether policies mentioned the potential to share user data with marketers or	2	Not specified; authors from: Department of Psychiatry (LR), Massachusetts General Hospital, Boston, MA; and Department of Psychiatry and Clinical Informatics (JT).	Descriptive

		advertisers, as evidenced by using any of these terms: “advertising, commercial purposes marketing, demographic profiling, industry analysis.” We also made note of whether user data might be sold in a merger or acquisition, or otherwise, and recorded whether policies admitted to potentially sharing identifiable data if legally bound. Finally, we reviewed each policy to see if individual-level data could be deleted or amended upon user request.<br>We noted whether each of these criteria was met, not met, or not mentioned in (i.e., was absent from) the privacy policy. Two of the authors (LR and JT) reviewed all policies using these criteria, and disagreements were resolved by consensus among all authors.			

59	Inclusion criteria: (1) Apps that have Italian-language user interfaces; (2) Apps used for blood glucose monitoring; (3)Apps designed to be used primarily by patients (whether with or without the intervention of health care professionals, such as doctors, nurses, etc.); (4) Apps designed (also) for patients with type 2 diabetes. Exclusion criteria: (1) Apps that have non-Italian user interfaces; (2) Apps designed to be used by doctors and/or other care professionals; (3) Apps that are not primarily designed for diabetes self-management (e.g., recipe apps; sport apps, such as pedometers); (4) Apps that are not specifically designed for diabetes; (5) Duplicated apps; (6) Paid apps; (7) Apps provided by healthcare facilities requiring login.	Testing protocol: Both authors of this study installed the apps and uploaded real life glycemic profiles for one month. The profiles do not correspond to the parameters of a single individual, but have been prepared in collaboration with an expert clinician. Finally, the authors observed the apps' responses by focusing on both verbal and visual dimensions. Assessment criteria: We analyzed the reported usability and functions of the 17 selected apps. Specifically, we analyzed apps with respect to their educational level, since empirical evidence and clinical guidelines stress the relevance of education for the improvement of diabetes self-management, a mediator of adherence.	2	Not specified; authors from: Department of Linguistic Sciences and Foreign Literatures, Catholic University of the Sacred Heart, Milano, Italy	Descriptive analysis of app functionalities and characteristics

60	Inclusion criteria: (1) apps that aimed to support medication self-management, (2) apps capable of generating scheduled reminders for medication-taking behavior, and (3) apps that were in English.	The apps were scored on practicality and functionality. The 5 apps with the highest score in each of the advanced and basic functionality categories were then used for the quality Mobile App Rating Scale (MARS) assessment.	1 for practicality and functionality; 2 for quality (MARS) assessment	The reviewers were trained to use the MARS instrument by watching an online tutorial to ensure that both reviewers used the tool in the same manner. One reviewer (KS) assessed the apps using an iOS device and the other reviewer (SR) used an Android device.	Descriptive: Percentages of how many apps met certain criteria. For the MARS quality assessment, they used the method outlined by MARS where the quality dimensions are given a score between 0 and 1.
61	Only English-language mHealth apps available in the UK market were selected following the original systematic search. Several criteria had to be considered while selecting apps from their respective markets. The exclusion criteria were: (1) Games for COPD patients; (2) Apps in different language; (3) Apps not available in UK market; (4) Apps with low ratings/no ratings; (5) Apps developed by individuals; and (6) Apps on how to use inhalers for COPD and asthma patients. The comprehensiveness and consistency of information were assessed for apps presenting health information about COPD.	Not specified; features of apps were recorded and reported.	Not specified	Not specified; authors from: Digital Media for Health, Medical Information and Network Technology, Faculty of Science, Engineering and Computing, Kingston University London, Surrey, United Kingdom; CIRO, Research and Education, Horn, Netherlands; and Pulmonary Clinic, George Papanikolaou General Hospital, Thessaloniki, Greece	Descriptive (and possibly thematic but this is unclear).

62	Apps designed for patients and/or health professionals and citing weight loss surgery were included whilst apps that did not focus specifically on surgical interventions were excluded (e.g. dietary apps, complementary/alternative treatment apps). Data were collected from the app overview provided by the developer and a purposive sample of free and paid apps were downloaded based on their intended use.	Recorded data included the cost ($ = US dollars), publisher information, number of ratings, average rating and any documentation of medical professional involvement. Apps were then subcategorised based on content topic and target audience. Results were tabulated using Excel 2010 (Microsoft, Redmond, WA, USA).	2	Not specified; authors from: North Bristol NHS Trust, Department of General Surgery, Southmead Hospital, Bristol, UK; Taunton and Somerset NHS Foundation Trust, Department of General Surgery, Musgrove Park Hospital, Somerset, UK; Department of Anaesthetics and Intensive Care, Bristol Royal Infirmary, University Hospitals Bristol NHS Trust, Bristol, UK	Thematic; Apps were subcategorized into themes and the researchers identified the target audience, number of apps, price range, apps that had customer satisfaction ratings available, average customer satisfaction rating and whether the apps had medical professional involvement.
63	Studies of Internet users have shown that most people rarely search beyond the first 20 retrieved results. However, we extended the coverage of the present study to the first 50 free apps and to the first 30 paid apps for each tag to obtain the most comprehensive list of apps. Apps were included if they were related to PD. Exclusion criteria were as follows: the app could not be downloaded after more than three attempts, the app was not in English, or the app was a book or an article.	Apps were reviewed on an HTC One Android 4.3. They were assessed by using tools reported in previous studies, tools adapted from quality evaluation studies of websites, and tools described in other studies on the quality of smartphone apps.	Not specified	Not specified; authors from: Geneva University, and Geneva University Hospitals Geneva, Switzerland	Statistical: An initial exploratory analysis involved the calculation of proportions, as well as means and SDs, of the above-mentioned outcome measures. Next, we compared paid apps with free apps in bivariate analyses by using parametric tests (t-test, chi-square, or Fisher’s exact test) or non-parametric tests (median test) when appropriate. Finally, we computed prediction models by using multiple linear regressions for two variables of interest.

64	The search initially yielded 602 apps. 312 apps were later excluded as the review of description indicated that they were irrelevant to the topic. A further review led to a removal of 139 duplicates. The final sample consisted of a total of 151 apps.	Each app was then downloaded onto a Huawei Galaxy 4X (Android) or i-phone 4S (iOS) smartphone and analyzed independently by two researchers. The contents of the top 5 downloaded apps were further reviewed against key topic areas as extracted from European Guidelines on cardiovascular disease prevention25 to determine if they have covered areas that were considered conventional.	2	Not specified; authors from: School of Nursing, Capital Medical University, Beijing, China.<br>College of Health and Biomedicine, Victoria University, Melbourne, Australia.	Data were analyzed using descriptive statistics. Numbers and percentages of apps with the respective features were calculated. The frequency, mean, and standard deviation were determined based on the Silberg assessment scale.
65	Applications were excluded if they had no clinical relevance (for example, if it was a gaming application or if it was an application that contains only advertisements).	The reviewers downloaded the 39 apps and used the Silberg Scale to produce a score.	2 (with 1 final assessor)	Two independent medical students, who were in their 2nd year of medical school at the National University of Singapore,assisted in the extraction of the relevant information and theinitial analysis of the scoring of the respective applications.Any disagreements with the scores were directed to a thirdindependent, assessor, (MWBZ) for final assessment. In addi-tion, the final assessor also checked through the individualapplications and re-computed the scores to make sure thatthe scores were valid.	The scores of the Silberg scale were analyzed using descriptive statistics. In addition to the Silberg scale, we also reported the totalnumber of users for the specific app and the respective appli-cation rating as indicators of usability.

Supplementary Table 3 - Search Strategy

MEDLINE Search History

#	Searches
1	Mobile Applications/
2	exp Cell Phones/
3	mhealth.ti,ab,kf.
4	mobile health.ti,ab,kf.
5	telehealth.ti,ab,kf.
6	ehealth.ti,ab,kf.
7	telehomecare.ti,ab,kf.
8	smartphone*.ti,ab,kf.
9	virtual care.ti,ab,kf.
10	android.ti,ab,kf.
11	ios.ti,ab,kf.
12	iphone.ti,ab,kf.
13	(mobile adj2 application*).ti,kf.
14	(mobile adj2 application*).ab.
15	exp Asthma/
16	(asthma or asthmas or asthmatic*).ti,ab,kf.
17	exp Mental Disorders/
18	(dementia praecox or schizophreni*).ti,ab,kf.
19	(bipolar or manic disorder* or mania or manias or manic state* or manic depressive or manic-depressive).ti,ab,kf.
20	(behavio$r disorder or mental disorder* or psychiatric diagnosis).ti,ab,kf.
21	Mentally Ill Persons/
22	(mental patients or mentally ill or mental illness).ti,ab,kf.
23	exp Cardiovascular Diseases/
24	(cardiovascular disease* or cardiac disease*).ti,ab,kf.
25	(High blood pressure or hypertension).ti,ab,kf.
26	Pulmonary Disease, Chronic Obstructive/
27	(Coad or copd or chronic airflow obstruction* or chronic obstructive airway disease or chronic obstructive lung disease or chronic obstructive pulmonary disease).ti,ab,kf.

28	(involutional depression or involutional melancholia or involutional paraphrenia* or involutional paraphrenias or involutional psychos$s or major depressive disorder*).ti,ab,kf.
29	exp Diabetes Mellitus/
30	(Diabetes or iddm or mody or niddm).ti,ab,kf.
31	medication adherence/
32	(Medication adj (adherence or compliance or non adherence or non-adherence or non-compliance or nonadherence or noncompliance or persistence)).ti,ab,kf.
33	patient compliance/
34	(Patient adj (adherence or compliance or cooperation or non-adherence or non-compliance or nonadherence or noncompliance)).ti,ab,kf.
35	Weight Loss/
36	Smoking Cessation/
37	smoking cessation.ti,ab,kf.
38	(smoking adj (cessation* or quit*)).ti,ab,kf.
39	Alcohol Drinking/
40	(Alcohol adj (consumption or drinking)).ti,ab,kf.
41	Weight Reduction Programs/
42	(Weight loss* or weight reduction*).ti,ab,kf.
43	Weight Loss/
44	or/31-43
45	search:.tw. or meta analysis.mp,pt. or review.pt. or di.xs. or associated.tw.
46	15 or 16 or 17 or 18 or 19 or 20 or 21 or 22 or 23 or 24 or 25 or 26 or 27 or 28 or 29 or 30
47	46 or 44
48	1 or 2 or 3 or 4 or 5 or 6 or 7 or 8 or 9 or 10 or 11 or 12 or 13 or 14
49	47 and 48
50	45 and 49

EMBASE Search History

#	Searches
1	mobile application/
2	exp mobile phone/
3	mhealth.ab,kw,ti.

4	mobile health.ti,ab,kw.
5	telehealth.ti,ab,kw.
6	ehealth.ti,ab,kw.
7	telehomecare.ti,ab,kw.
8	smartphone*.ti,ab,kw.
9	virtual care.ti,ab,kw.
10	android.ti,ab,kw.
11	ios.ti,ab,kw.
12	iphone.ti,ab,kw.
13	(mobile adj2 application*).ti,kw.
14	(mobile adj2 application*).ab.
15	exp asthma/
16	(asthma or asthmas or asthmatic*).ti,ab,kw.
17	exp mental disease/
18	(dementia praecox or schizophreni*).ti,ab,kw.
19	(bipolar or manic disorder* or mania or manias or manic state* or manic depressive or manic-depressive).ti,ab,kw.
20	(behavio$r disorder or mental disorder* or psychiatric diagnosis).ti,ab,kw.
21	mental patient/
22	(mental patients or mentally ill or mental illness).ti,ab,kw.
23	exp cardiovascular disease/
24	(cardiovascular disease* or cardiac disease*).ti,ab,kw.
25	(High blood pressure or hypertension).ti,ab,kw.
26	chronic obstructive lung disease/
27	(Coad or copd or chronic airflow obstruction* or chronic obstructive airway disease or chronic obstructive lung disease or chronic obstructive pulmonary disease).ti,ab,kw.
28	(involutional depression or involutional melancholia or involutional paraphrenia* or involutional paraphrenias or involutional psychos$s or major depressive disorder*).ti,ab,kw.
29	exp diabetes mellitus/
30	(Diabetes or iddm or mody or niddm).ti,ab,kw.
31	(Medication adj (adherence or compliance or non adherence or non-adherence or non-compliance or nonadherence or noncompliance or persistence)).ti,ab,kw.
32	exp patient compliance/

33	(Patient adj (adherence or compliance or cooperation or non-adherence or non-compliance or nonadherence or noncompliance)).ti,ab,kw.
34	weight reduction/
35	smoking cessation/ or smoking cessation program/
36	smoking cessation.ti,ab,kw.
37	(smoking adj (cessation* or quit*)).ti,ab,kw.
38	drinking behavior/
39	(Alcohol adj (consumption or drinking)).ti,ab,kw.
40	weight loss program/
41	(Weight loss* or weight reduction*).ti,ab,kw.
42	or/31-41
43	exp methodology/ or search:.tw. or review.pt.
44	15 or 16 or 17 or 18 or 19 or 20 or 21 or 22 or 23 or 24 or 25 or 26 or 27 or 28 or 29 or 30
45	42 or 44
46	1 or 2 or 3 or 4 or 5 or 6 or 7 or 8 or 9 or 10 or 11 or 12 or 13 or 14
47	45 and 46
48	43 and 47

Cochrane Central Database of Controlled Trials Search Strategy

#	Searches
1	MeSH descriptor: [Mobile Applications] explode all trees
2	MeSH descriptor: [Cell Phones] explode all trees
3	mhealth or "mobile health" or telehealth or ehealth or telehomecare or smartphone* or "virtual care" or android or ios or iphone*:ti,ab,kw (Word variations have been searched)
4	mobile near/2 application*:ti,ab,kw (Word variations have been searched)
5	(or #1-#4)
6	MeSH descriptor: [Asthma] explode all trees
7	asthma or asthmas or asthmatic:ti,ab,kw (Word variations have been searched)
8	MeSH descriptor: [Mental Disorders] explode all trees
9	"dementia praecox" or schizophreni*:ti,ab,kw (Word variations have been searched)
10	bipolar or (manic next disorder*) or mania or manias or (manic next state*) or "manic depressive" or "manic-depressive":ti,ab,kw (Word variations have been searched)
11	((behavior or behaviour or mental) next disorder) or "psychiatric diagnosis":ti,ab,kw (Word variations have been searched)
12	MeSH descriptor: [Mentally Ill Persons] explode all trees

13	"mental patients" or "mentally ill" or "mental illness":ti,ab,kw (Word variations have been searched)
14	MeSH descriptor: [Cardiovascular Diseases] explode all trees
15	((cardiovascular or cardiac) next disease*):ti,ab,kw (Word variations have been searched)
16	"high blood pressure" or hypertension:ti,ab,kw (Word variations have been searched)
17	MeSH descriptor: [Pulmonary Disease, Chronic Obstructive] explode all trees
18	coad or copd or ("chronic airflow" next obstruction*) or "chronic obstructive airway disease" or "chronic obstructive lung disease" or "chronic obstructive pulmonary disease":ti,ab,kw (Word variations have been searched)
19	(involutional next (depression or melancholia or paraphenia* or psychosis or psychoses)) or ("major depressive" next disorder*):ti,ab,kw (Word variations have been searched)
20	MeSH descriptor: [Diabetes Mellitus] explode all trees
21	diabetes or iddm or mody or niddm:ti,ab,kw (Word variations have been searched)
22	MeSH descriptor: [Medication Adherence] explode all trees
23	medication next (adherence or compliance or cooperation or "non-adherence" or "non-compliance" or nonadherence or noncompliance or persistence):ti,ab,kw (Word variations have been searched)
24	MeSH descriptor: [Patient Compliance] explode all trees
25	patient next (adherence or compliance or cooperation or "non-adherence" or "non-compliance" or nonadherence or noncompliance):ti,ab,kw (Word variations have been searched)
26	MeSH descriptor: [Weight Loss] explode all trees
27	MeSH descriptor: [Weight Reduction Programs] explode all trees
28	weight next (loss* or reduction*):ti,ab,kw (Word variations have been searched)
29	MeSH descriptor: [Smoking Cessation] explode all trees
30	smoking next (cessation* or quit*):ti,ab,kw (Word variations have been searched)
31	MeSH descriptor: [Alcohol Drinking] explode all trees
32	alcohol next (drinking or consumption):ti,ab,kw (Word variations have been searched)
33	(or #6-#32)
34	#5 and #33

References 1 – References of Included Articles

1 Abroms, L. C., Westmaas, J. L., Bontemps-Jones, J., Ramani, R. & Mellerson, J. A content analysis of popular smartphone apps for smoking cessation. *American journal of preventive medicine* **45**, 732-736 (2013).

2 Abroms, L. C., Padmanabhan, N., Thaweethai, L. & Phillips, T. iPhone apps for smoking cessation: a content analysis. *American journal of preventive medicine* **40**, 279-285 (2011).

3 Alhuwail, D. in *Nursing Informatics.* 587-591.

4 Alnasser, A. A., Amalraj, R. E., Sathiaseelan, A., Al-Khalifa, A. S. & Marais, D. Do Arabic weight-loss apps adhere to evidence-informed practices? *Translational behavioral medicine* **6**, 396-402 (2016).

5 Arnhold, M., Quade, M. & Kirch, W. Mobile applications for diabetics: a systematic review and expert-based usability evaluation considering the special requirements of diabetes patients age 50 years or older. *Journal of medical Internet research* **16**, e104 (2014).

6 Bardus, M., van Beurden, S. B., Smith, J. R. & Abraham, C. A review and content analysis of engagement, functionality, aesthetics, information quality, and change techniques in the most popular commercial apps for weight management. *International Journal of Behavioral Nutrition and Physical Activity* **13**, 35 (2016).

7 Basilico, A., Marceglia, S., Bonacina, S. & Pinciroli, F. Advising patients on selecting trustful apps for diabetes self-care. *Computers in biology and medicine* **71**, 86-96 (2016).

8 Breland, J. Y., Yeh, V. M. & Yu, J. Adherence to evidence-based guidelines among diabetes self-management apps. *Translational behavioral medicine* **3**, 277-286 (2013).

9 Brzan, P. P., Rotman, E., Pajnkihar, M. & Klanjsek, P. Mobile applications for control and self management of diabetes: a systematic review. *Journal of medical systems* **40**, 210 (2016).

10 Cuenca, M. R. C., Cuenca, M. D. C. & Verdugo, R. M. Availability and medical professional involvement in mobile healthcare applications related to pathophysiology and pharmacotherapy of HIV/AIDS. *European Journal of Hospital Pharmacy: Science and Practice* **20**, 356-361 (2013).

11 Carter, T., O'Neill, S., Johns, N. & Brady, R. R. Contemporary vascular smartphone medical applications. *Annals of vascular surgery* **27**, 804-809 (2013).

12 Choi, J., Noh, G.-Y. & Park, D.-J. Smoking cessation apps for smartphones: content analysis with the self-determination theory. *Journal of medical Internet research* **16**, e44 (2014).

13 Chomutare, T., Fernandez-Luque, L., Årsand, E. & Hartvigsen, G. Features of mobile diabetes applications: review of the literature and analysis of current applications compared against evidence-based guidelines. *Journal of medical Internet research* **13**, e65 (2011).

14 Cohn, A. M., Hunter-Reel, D., Hagman, B. T. & Mitchell, J. Promoting behavior change from alcohol use through mobile technology: the future of ecological momentary assessment. *Alcoholism: Clinical and Experimental Research* **35**, 2209-2215 (2011).

15 Crane, D., Garnett, C., Brown, J., West, R. & Michie, S. Behavior change techniques in popular alcohol reduction apps: content analysis. *Journal of medical Internet research* **17**, e118 (2015).

16 Darby, A., Strum, M. W., Holmes, E. & Gatwood, J. A review of nutritional tracking mobile applications for diabetes patient use. *Diabetes technology & therapeutics* **18**, 200-212 (2016).

17 Dayer, L., Heldenbrand, S., Anderson, P., Gubbins, P. O. & Martin, B. C. Smartphone medication adherence apps: potential benefits to patients and providers. *Journal of the American Pharmacists Association* **53**, 172-181 (2013).

18 Demidowich, A. P., Lu, K., Tamler, R. & Bloomgarden, Z. An evaluation of diabetes self-management applications for Android smartphones. *Journal of telemedicine and telecare* **18**, 235-238 (2012).

19 Drincic, A., Prahalad, P., Greenwood, D. & Klonoff, D. C. Evidence-based mobile medical applications in diabetes. *Endocrinology and Metabolism Clinics* **45**, 943-965 (2016).

20 Dubey, D. *et al.* Smart phone applications as a source of information on stroke. *Journal of stroke* **16**, 86 (2014).
21 El-Gayar, O., Timsina, P., Nawar, N. & Eid, W. Mobile applications for diabetes self-management: status and potential. *Journal of diabetes science and technology* **7**, 247-262 (2013).
22 Fairburn, C. G. & Rothwell, E. R. Apps and eating disorders: A systematic clinical appraisal. *International Journal of Eating Disorders* **48**, 1038-1046 (2015).
23 Formagini, T. D. B. *et al.* A review of smartphone apps for smoking cessation available in Portuguese. *Cadernos de saude publica* **33**, e00178215 (2017).
24 Franco, R. Z., Fallaize, R., Lovegrove, J. A. & Hwang, F. Popular nutrition-related mobile apps: a feature assessment. *JMIR mHealth and uHealth* **4**, e85 (2016).
25 Gao, C., Zhou, L., Liu, Z., Wang, H. & Bowers, B. Mobile application for diabetes self-management in China: Do they fit for older adults? *International journal of medical informatics* **101**, 68-74 (2017).
26 Haase, J., Farris, K. B. & Dorsch, M. P. Mobile applications to improve medication adherence. *Telemedicine and e-Health* **23**, 75-79 (2017).
27 Hale, K., Capra, S. & Bauer, J. A framework to assist health professionals in recommending high-quality apps for supporting chronic disease self-management: illustrative assessment of type 2 diabetes apps. *JMIR mHealth and uHealth* **3**, e87 (2015).
28 Hales, S., Dunn, C., Wilcox, S. & Turner-McGrievy, G. M. Is a picture worth a thousand words? Few evidence-based features of dietary interventions included in photo diet tracking mobile apps for weight loss. *Journal of diabetes science and technology* **10**, 1399-1405 (2016).
29 Haskins, B. L., Lesperance, D., Gibbons, P. & Boudreaux, E. D. A systematic review of smartphone applications for smoking cessation. *Translational behavioral medicine* **7**, 292-299 (2017).
30 Heldenbrand, S. *et al.* Assessment of medication adherence app features, functionality, and health literacy level and the creation of a searchable Web-based adherence app resource for health care professionals and patients. *Journal of the American Pharmacists Association* **56**, 293-302 (2016).
31 Hoeppner, B. B. *et al.* How smart are smartphone apps for smoking cessation? A content analysis. *Nicotine & Tobacco Research* **18**, 1025-1031 (2016).
32 Hoppe, C. D., Cade, J. E. & Carter, M. An evaluation of diabetes targeted apps for Android smartphone in relation to behaviour change techniques. *Journal of human nutrition and dietetics* **30**, 326-338 (2017).
33 Househ, M. *et al.* A cross-sectional content analysis of Android applications for asthma. *Health informatics journal* **23**, 83-95 (2017).
34 Huckvale, K., Adomaviciute, S., Prieto, J. T., Leow, M. K.-S. & Car, J. Smartphone apps for calculating insulin dose: a systematic assessment. *BMC medicine* **13**, 106 (2015).
35 Huckvale, K., Car, M., Morrison, C. & Car, J. Apps for asthma self-management: a systematic assessment of content and tools. *BMC medicine* **10**, 144 (2012).
36 Huguet, A. *et al.* A systematic review of cognitive behavioral therapy and behavioral activation apps for depression. *PloS one* **11** (2016).
37 Issom, D.-Z. *et al.* Mobile applications for people with diabetes published between 2010 and 2015. *Diabetes Management* **5**, 539-550 (2015).
38 Jacobs, M. A., Cobb, C. O., Abroms, L. & Graham, A. L. Facebook apps for smoking cessation: a review of content and adherence to evidence-based guidelines. *Journal of medical Internet research* **16**, e205 (2014).
39 Jeon, E., Park, H., Min, Y. H. & Kim, H.-Y. Analysis of the information quality of Korean obesity-management smartphone applications. *Healthcare informatics research* **20**, 23-29 (2014).
40 Juarascio, A. S., Manasse, S. M., Goldstein, S. P., Forman, E. M. & Butryn, M. L. Review of smartphone applications for the treatment of eating disorders. *European Eating Disorders Review* **23**, 1-11 (2015).

41 Kumar, N., Khunger, M., Gupta, A. & Garg, N. A content analysis of smartphone–based applications for hypertension management. *Journal of the American Society of Hypertension* **9**, 130-136 (2015).
42 Kumar, S. & Mehrotra, S. Free mobile apps on depression for Indian users: A brief overview and critique. *Asian journal of psychiatry* **28**, 124-130 (2017).
43 Larsen, M. E., Nicholas, J. & Christensen, H. A systematic assessment of smartphone tools for suicide prevention. *PloS one* **11** (2016).
44 Martínez-Pérez, B., De La Torre-Díez, I., López-Coronado, M. & Herreros-González, J. Mobile apps in cardiology. *JMIR mHealth and uHealth* **1**, e15 (2013).
45 Martínez-Pérez, B., De La Torre-Díez, I. & López-Coronado, M. Mobile health applications for the most prevalent conditions by the World Health Organization: review and analysis. *Journal of medical Internet research* **15**, e120 (2013).
46 Modave, F. *et al.* Low quality of free coaching apps with respect to the American College of Sports Medicine guidelines: a review of current mobile apps. *JMIR mHealth and uHealth* **3**, e77 (2015).
47 Moodley, A., Mangino, J. E. & Goff, D. A. Review of infectious diseases applications for iPhone/iPad and Android: from pocket to patient. *Clinical infectious diseases* **57**, 1145-1154 (2013).
48 Morrissey, E. C., Corbett, T. K., Walsh, J. C. & Molloy, G. J. Behavior change techniques in apps for medication adherence: a content analysis. *American journal of preventive medicine* **50**, e143-e146 (2016).
49 Myint, M., Adam, A., Herath, S. & Smith, G. Mobile phone applications in management of enuresis: the good, the bad, and the unreliable! *Journal of pediatric urology* **12**, 112. e111-112. e116 (2016).
50 Nguyen, A. D. *et al.* Mobile applications to enhance self-management of gout. *International journal of medical informatics* **94**, 67-74 (2016).
51 Nguyen, E. *et al.* Is there a good app for that? Evaluating m-Health apps for strategies that promote pediatric medication adherence. *Telemedicine and e-Health* **22**, 929-937 (2016).
52 Nicholas, J., Larsen, M. E., Proudfoot, J. & Christensen, H. Mobile apps for bipolar disorder: a systematic review of features and content quality. *Journal of medical Internet research* **17**, e198 (2015).
53 Nikolaou, C. K. & Lean, M. E. Mobile applications for obesity and weight management: current market characteristics. *International Journal of Obesity* **41**, 200-202 (2017).
54 Pagoto, S., Schneider, K., Jojic, M., DeBiasse, M. & Mann, D. Evidence-based strategies in weight-loss mobile apps. *American journal of preventive medicine* **45**, 576-582 (2013).
55 Patel, R. *et al.* Smartphone apps for weight loss and smoking cessation: quality ranking of 120 apps. *NZ Med J* **128**, 73-76 (2015).
56 Penzenstadler, L., Chatton, A., Van Singer, M. & Khazaal, Y. Quality of smartphone apps related to alcohol use disorder. *European addiction research* **22**, 329-338 (2016).
57 Radovic, A. *et al.* Smartphone applications for mental health. *Cyberpsychology, Behavior, and Social Networking* **19**, 465-470 (2016).
58 Rosenfeld, L., Torous, J. & Vahia, I. V. Data security and privacy in apps for dementia: an analysis of existing privacy policies. *The American Journal of Geriatric Psychiatry* **25**, 873-877 (2017).
59 Rossi, M. G. & Bigi, S. mHealth for diabetes support: a systematic review of apps available on the Italian market. *Mhealth* **3** (2017).
60 Santo, K. *et al.* Mobile phone apps to improve medication adherence: a systematic stepwise process to identify high-quality apps. *JMIR mHealth and uHealth* **4**, e132 (2016).
61 Sobnath, D. D. *et al.* Features of a mobile support app for patients with chronic obstructive pulmonary disease: literature review and current applications. *JMIR mHealth and uHealth* **5**, e17 (2017).

62 Stevens, D. J., Jackson, J. A., Howes, N. & Morgan, J. Obesity surgery smartphone apps: a review. *Obesity surgery* **24**, 32-36 (2014).

64 Van Singer, M., Chatton, A. & Khazaal, Y. Quality of smartphone apps related to panic disorder. *Frontiers in psychiatry* **6**, 96 (2015).

64 Xiao, Q., Lu, S., Wang, Y., Sun, L. & Wu, Y. Current status of cardiovascular disease-related smartphone apps downloadable in China. *Telemedicine and e-Health* **23**, 219-225 (2017).

65 Zhang, M. W., Ho, R. C., Hawa, R. & Sockalingam, S. Analysis of the information quality of bariatric surgery smartphone applications using the silberg scale. Obesity surgery 26, 163-168 (2016).
